# Development and Characterization of Functionally Graded Sisal/Glass Fiber-Reinforced Polymer Composites

**DOI:** 10.3390/ma19153295

**Published:** 2026-08-03

**Authors:** Henok Dereje Nega, Hailu Shimels Gebremedhen, Tomasz Trzepieciński, Fasikaw Kibrete, Temesgen Tadesse Feisa, Dereje Engida Woldemichael

**Affiliations:** 1 Department of Mechanical Engineering, College of Engineering, Addis Ababa Science and Technology University, Addis Ababa P.O. Box 16417, Ethiopia; henok.derejen@aastustudent.edu.et (H.D.N.); dereje.engida@aastu.edu.et (D.E.W.); 2 Université Marie et Louis Pasteur, UTBM, CNRS, ICB UMR 6303, 90010 Belfort, France; hailu.gebremedhen@utbm.fr; 3 Faculty of Mechanical Engineering and Aeronautics, Rzeszów University of Technology, al. Powstańców Warszawy 8, 35-029 Rzeszów, Poland; 4 Department of Mechanical Engineering, Institute of Technology, University of Gondar, Gondar P.O. Box 196, Ethiopia; fasikaw.kibrete@uog.edu.et; 5 Department of Mechanical Engineering, College of Engineering and Technology, Dilla University, Dilla P.O. Box 419, Ethiopia; 6 Artificial Intelligence and Robotic Center of Excellence, Addis Ababa Science and Technology University, Addis Ababa P.O. Box 16417, Ethiopia

**Keywords:** functionally graded composites, sisal fiber, glass fiber, polymer matrix composites, structural applications, sustainable materials

## Abstract

Structural applications require materials that provide an optimal balance of strength, durability, and sustainability. This study develops functionally graded sisal/glass fiber-reinforced polymer composites and evaluates their tensile, compressive, and flexural properties, as well as their water absorption behavior. First, an experimental approach was adopted in which sisal fibers were treated with a 6% sodium hydroxide (NaOH) solution to improve fiber–matrix adhesion. The composites were fabricated using the hand lay-up method with varying sisal fiber ratios. Mechanical testing showed that the composites achieved a tensile strength of 146 MPa for the rectangular specimens and 199.5 MPa for the composite bars. The highest compressive strength (120 MPa) and flexural strength (326 MPa) were observed at 20% sisal fiber content. In addition, finite element analysis (FEA) results showed maximum tensile and compressive strengths of 181.24 MPa and 147.57 MPa, respectively, and were in reasonable agreement with the experimental results. Moreover, water absorption studies showed a direct correlation between fiber content and moisture uptake, with values of 1.40% (10% sisal), 2.55% (20%), and 3.85% (30%). The results indicate that functionally graded sisal/glass fiber-reinforced composites exhibit superior mechanical properties compared with conventional composites. The graded structure enhances design flexibility, optimizing material performance for structural applications. These findings highlight the potential of functionally graded composites as a sustainable alternative that addresses the limitations of traditional materials while promoting more efficient and environmentally friendly engineering solutions.

## 1. Introduction

In recent years, the construction industry has experienced substantial growth and has become a major contributor to the global economy. Statistics show that the construction industry contributes approximately 13% to the world’s GDP [1]. This growth is driven by factors such as rapid urbanization, infrastructure expansion, and global population growth [2]. The growing demand for modern infrastructure and sustainable construction has accelerated the development of innovative structural materials worldwide.

As a result, the construction sector requires lightweight, cost-effective, and durable materials capable of withstanding various environmental conditions and loading scenarios [3]. The demand for structural materials capable of offering extended service life while requiring minimal maintenance has led to a heightened interest in advanced composite materials for structural reinforcement. These materials are increasingly favored in new construction endeavors and the renovation of existing structures, where their promising properties and versatility offer significant advantages over conventional building materials like pure metals, alloys, and traditional composites.

Functionally graded materials (FGMs) have recently emerged as an important class of advanced composite materials [4]. These composites feature a continuous or stepwise variation in the composition of two or more components, optimizing their performance for specific applications. FGMs were initially introduced in 1984 by Japanese materials scientists as a way to alleviate thermal stress at metal-ceramic interfaces [5]. Since their introduction, FGMs have attracted considerable research interest because of their potential to enhance structural performance.

Researchers have developed a wide variety of FGMs, encompassing metals, ceramics, and polymers, to establish specific property gradients within the material structure. In particular, polymer-based FGMs have garnered significant attention in contemporary research due to their lightweight characteristics, affordability, and ease of production [6]. This trend highlights the growing recognition of polymer-based FGMs as versatile materials offering advantages in design flexibility, performance optimization, and manufacturing efficiency.

Among FGMs, natural fiber-based composites have attracted increasing attention because of their sustainability and favorable specific mechanical properties [7]. Natural fibers, primarily made of cellulose, offer a sustainable, plant-derived resource, whereas animal fibers are distinguished by their protein composition. The growing adoption of these materials is driven by their cost-effectiveness, recyclability, non-toxic properties, and favorable strength-to-weight ratios compared to synthetic alternatives. These environmentally friendly composites are manufactured by integrating natural fibers with various matrices to produce materials that are not only sustainable but also exhibit remarkable mechanical properties.

To enhance the mechanical performance and achieve a homogeneous composite structure, advanced composite design and fabrication methods are essential. Techniques such as optimized fiber orientation, surface modification of fibers, and the application of compatibilizers can improve the interfacial bonding and overall mechanical properties of the composites [8,9]. Furthermore, state-of-the-art methods such as resin infusion and 3D printing are being utilized to develop complex geometries and tailored material properties, facilitating the effective combination of natural and synthetic fibers [10].

However, the hydrophilic properties of natural fibers can diminish composite performance because of moisture absorption [11,12]. To address this challenge, hybridization with man-made fibers, like glass fibers, can enhance the overall performance of the composites [13]. By combining natural fibers with synthetic counterparts, specifically sisal and glass fibers, the resulting FGMs are anticipated to exhibit improved mechanical properties and greater environmental benefits than those achieved by any single reinforcement type. This approach combines the advantages of natural and synthetic fibers, resulting in a composite with improved mechanical performance and enhanced sustainability.

The rationale behind this paper lies in the potential of hybridizing synthetic and natural fibers in FGMs to achieve advantageous mechanical properties for structural applications. These hybrid composites are positioned to significantly reduce environmental impact compared to conventional counterparts. This research is motivated by the potential of these materials to make positive contributions to environmental sustainability and structural performance, thus paving the way for an eco-friendly future in construction.

The subsequent sections of this paper are organized as follows: Section 2 details the materials and methods used, Section 3 presents the results and discussion, and Section 4 concludes the paper and provides future perspectives.

## 2. Materials and Methods

**Figure 1:**
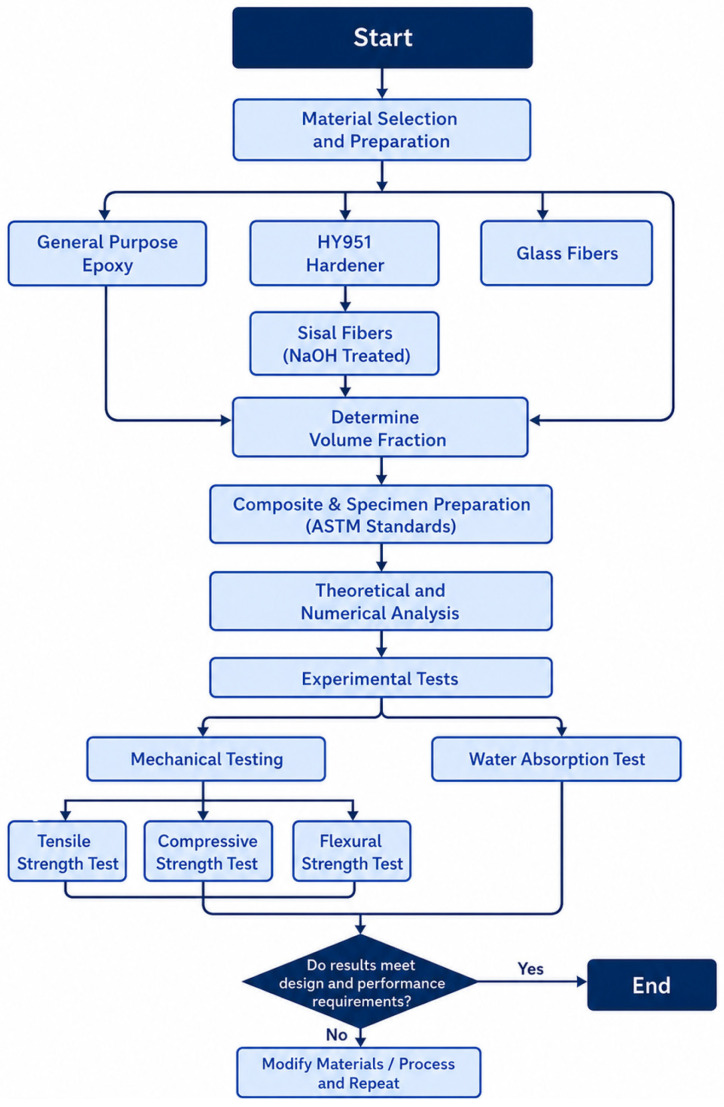
Flowchart of the research methodology.

This section describes the materials used to fabricate the functionally graded sisal/glass fiber-reinforced polymer composites and the experimental procedures employed to evaluate their mechanical properties and water absorption characteristics. The overall research method is presented in Figure 1 and follows a systematic approach to identify suitable materials and methods. The selected materials include general-purpose epoxy, HY951 hardener, and reinforcing fibers such as glass and sisal, with the sisal fibers undergoing sodium hydroxide (NaOH) treatment to enhance their properties. Based on the calculated volume fractions, the constituent materials were prepared and fabricated according to ASTM standards. The experimental evaluations included mechanical tests, specifically tensile, compressive, and flexural strength assessments, along with water absorption analysis. The results were analyzed and benchmarked against state-of-the-art materials for future advancements in the field.

### 2.1. Materials

The functionally graded sisal/glass fiber-reinforced polymer composites developed in this study are made up of E-glass fibers, sisal fibers, general-purpose epoxy, and a hardener. The glass fibers were sourced from World Fiber Glass and Waterproofing Engineering Ltd. in Addis Ababa, Ethiopia. The properties of the glass fiber are summarized in Table 1.

**Figure 2:**
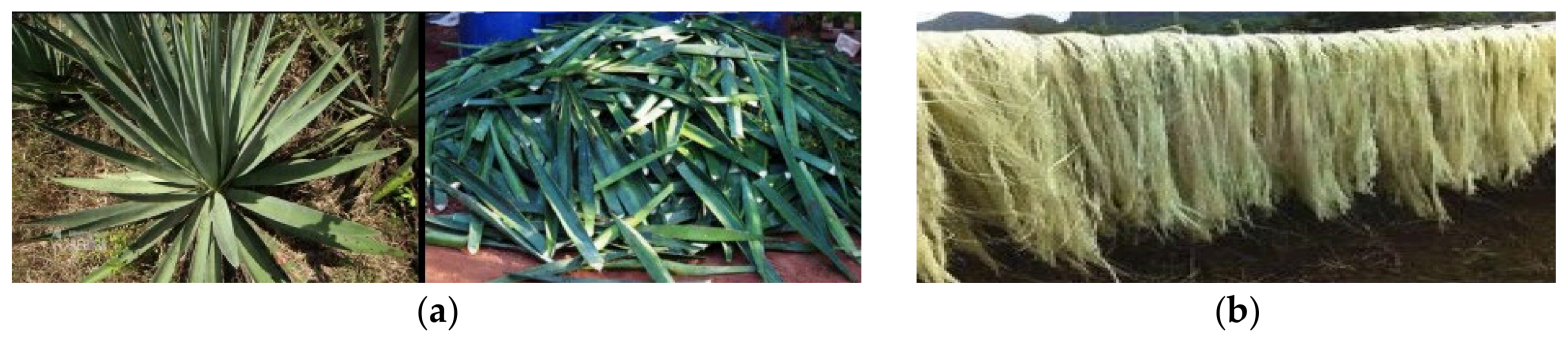
Fabrication of sisal fiber: (**a**) Sisal plant leaves; (**b**) Untreated sisal fibers.

The leaves of the *Agave sisalana* plant were manually processed to extract raw sisal fibers, which are widely recognized as sustainable reinforcement materials due to their favorable mechanical properties. Composed mainly of cellulose, sisal fibers exhibit significant tensile strength and stiffness, making them a potential alternative to synthetic fibers [14]. However, challenges such as poor compatibility with polymer matrices, susceptibility to UV degradation, and thermal sensitivity raise concerns about the long-term durability of sisal fiber-reinforced composites. Despite these issues, the high strength-to-weight ratio of sisal fibers makes them attractive for engineering applications [15]. Sisal fibers were selected because of their renewability, biodegradability, and favorable specific mechanical properties. Figure 2 shows the leaves and the untreated sisal fibers, showcasing the initial stages of this sustainable material preparation process.

**Figure 3:**
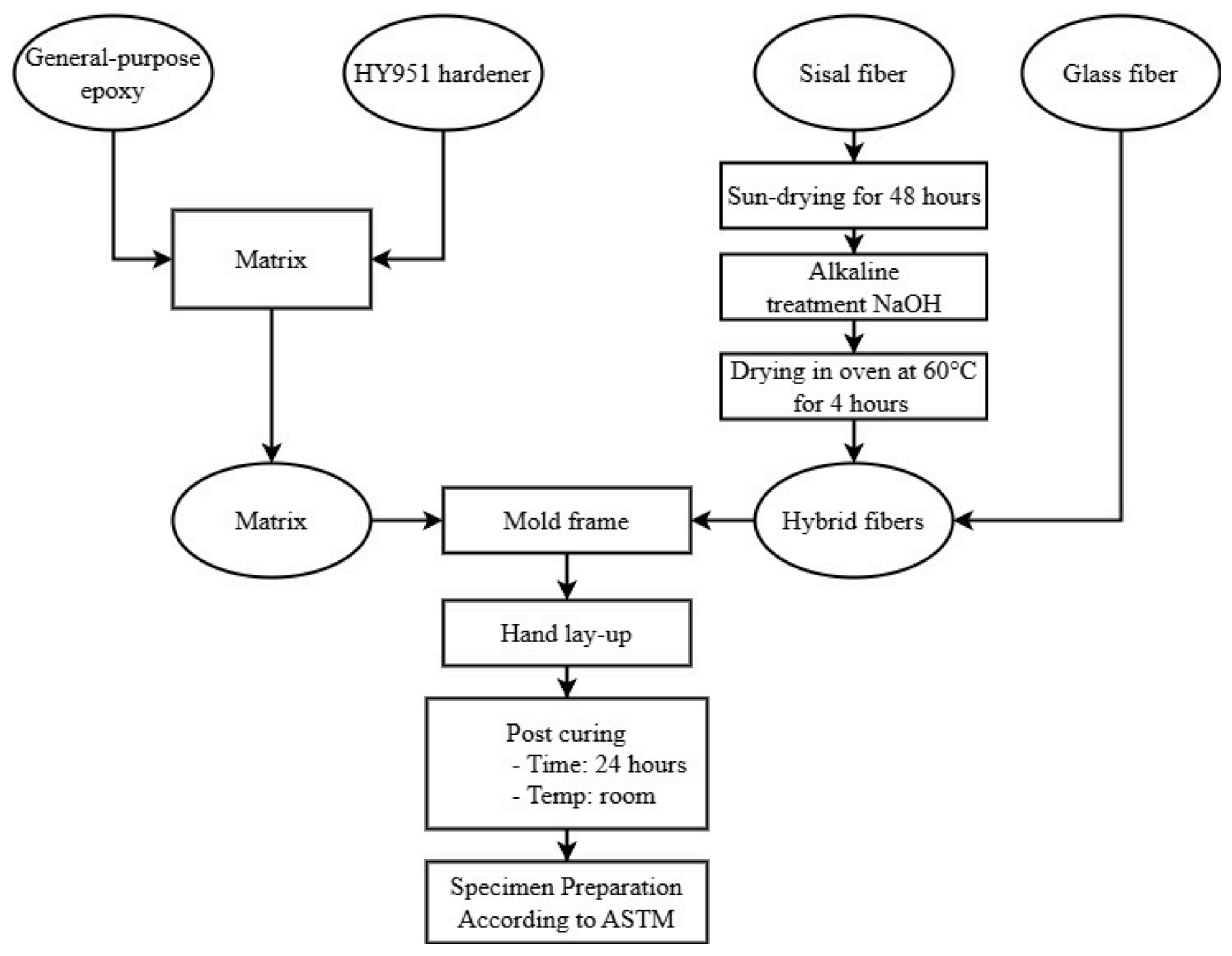
Schematic flowchart of the composite fabrication process.

The raw sisal fibers were then washed with distilled water. After washing, the sisal fibers were sun-dried for 48 h to remove any unbound water. Once dried, these fibers were labeled as untreated fibers and were prepared for alkali surface treatment. Figure 3 shows the flowchart of the fabrication process. The treatment process and the composite preparation are detailed in Section 2.2 and Section 2.3, respectively.

In this paper, a general-purpose epoxy resin was used as the polymer matrix for the composites because of its durability and versatility. This type of resin is particularly suitable for hand lay-up. The epoxy resin was sourced from a local supplier. The characteristics of the epoxy and hardener are presented in Table 2.

### 2.2. Alkaline Treatment of Sisal Fibers

In the present study, sisal fibers underwent an alkaline treatment to remove lignin and hemicellulose using NaOH. The treatment removed surface impurities and increased surface roughness, thereby improving fiber–matrix adhesion [16]. The treatment also increased the surface area available for bonding, which is crucial for achieving strong interfacial adhesion [17].

The treatment process involved immersing the fibers in a 6% NaOH solution at room temperature for 3 h, based on the recommendations provided in studies. During this process, a chemical reaction occurred between the hydroxyl groups (–OH) in the fibers and NaOH, resulting in the formation of a chemical bond, which is expressed as:

(1) $$\left(Fiber\right)-\left(OH\right)+\left(NaOH\right)\to (Fiber)-O-Na+H_{2}O,$$

**Figure 4:**
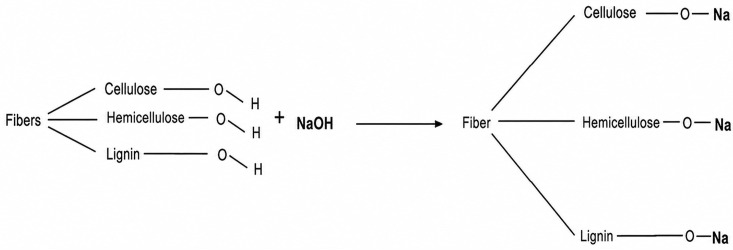
Interaction between NaOH and –OH groups in natural fibers.

Figure 4 shows the reaction between NaOH and the –OH groups. This chemical bond formation significantly improves the adhesion between the fibers and the epoxy polymer matrix.

After treatment, the fibers were thoroughly rinsed with distilled water to eliminate any remaining NaOH solution, ensuring that no excess alkaline residue lingered on the fiber surfaces. Finally, the treated fibers were allowed to air dry at room temperature for another 24 h before being utilized in the composite fabrication process.

The physical and mechanical properties of the treated sisal fibers are summarized in Table 3. These properties were obtained from the literature [18] and reflect the characteristics of sisal fibers treated with 6% NaOH.

### 2.3. Fabrication Method

**Figure 5:**
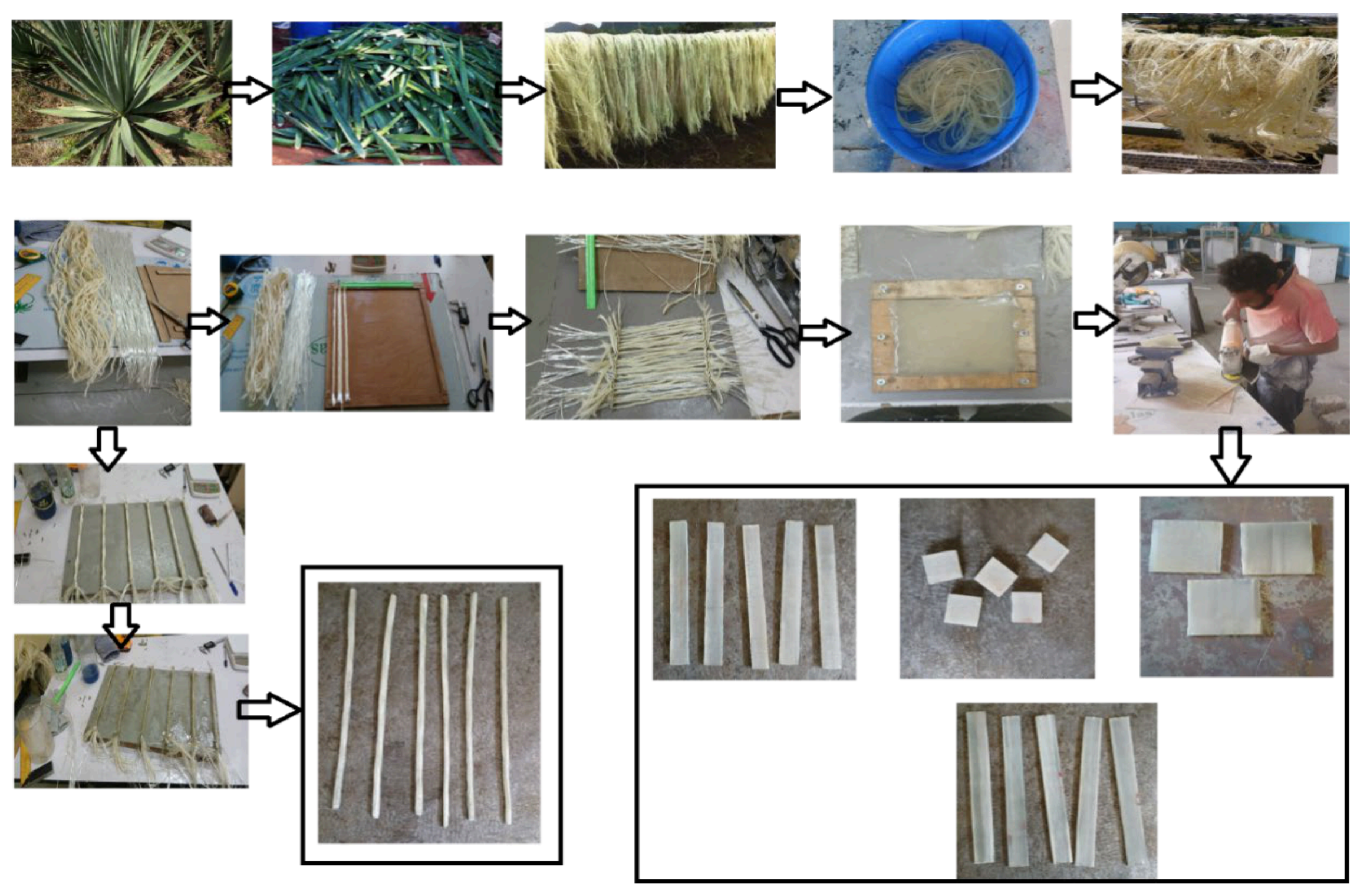
Overall composite fabrication process.

In this paper, the developed composites were produced via the hand lay-up technique. This conventional technique was selected because of its simplicity, ease of processing, and cost-effectiveness. The overall fabrication process is depicted in Figure 5.

Composite fabrication involved several steps, starting with the extraction and processing of sisal fibers. These sisal fibers were manually separated from the leaves using a hand-operated technique and thoroughly rinsed with distilled water to remove contaminants. The fibers were then subjected to a 3 h NaOH treatment. Alkaline treatment was applied based on previous studies reporting that it can remove surface impurities, reduce non-cellulosic components, and improve fiber–matrix interaction in natural fiber composites. However, the chemical and morphological changes induced by the treatment were not experimentally characterized in the present study.

Following this treatment, the fibers were rinsed multiple times with distilled water to neutralize any residual NaOH and subsequently oven-dried at 60 °C for 4 h to remove any remaining moisture. Lastly, the dried fibers were left to air-dry at ambient temperature for 24 h before being incorporated into the composite fabrication process.

Once dried, the fibers were combed to separate the strands and create a slightly rough surface, improving their bonding with the matrix. Both glass and sisal fibers were prepared according to the predetermined fiber ratio using an electronic weighing scale. A mold measuring 300 mm × 300 mm made of plywood was prepared and coated with beeswax to facilitate easy separation after curing. The fabrication process involved producing a stepwise functionally graded composite laminate, beginning with the impregnation of glass fibers with the matrix and layering them onto the mold. Next, a hybrid layer of 50% glass and 50% sisal fibers, also impregnated with the matrix, was applied. Finally, the sisal fibers were impregnated with the matrix and placed to complete the stepped gradient structure. All fibers were oriented at 0° to maintain uniform mechanical properties. Once all the layers were impregnated and arranged, the matrix was poured to fill the remaining gaps, and the mold was covered. The same fabrication procedure was used for all composite configurations, with only the sisal fiber content being varied. The composites were left to cure for 24 h before being cut into the desired shape using an angle grinder.

The fabricated laminates represent a stepwise functionally graded composite, in which the reinforcement composition changes discretely rather than continuously through the laminate thickness. Specifically, the laminate consists of a symmetric five-layer architecture comprising glass fiber, hybrid glass–sisal, sisal, hybrid glass–sisal, and glass fiber layers. This layered configuration provides a gradual transition in reinforcement composition and corresponding mechanical properties across the laminate thickness through successive discrete layers while remaining compatible with conventional hand lay-up fabrication. The layer-wise fiber compositions and corresponding volume fractions are presented in Section 3.1.1.

For the fabrication of composite bars (6 mm FRP bar), the fibers were impregnated and suspended between two fixed poles using a rope manufacturing technique. Initially, the sisal fibers were impregnated with the matrix, pulled out, and hung between the poles to form the inner core. A hybrid layer of 50% glass and 50% sisal fibers, impregnated with the matrix, was applied over the inner core. Finally, the outermost layer consisted of glass fibers impregnated with the matrix, covering the entire composite. The matrix was then poured over the fibers to ensure complete encapsulation. The bars were left to cure for 24 h before being cut into the desired shape using an angle grinder.

To improve the reliability of the investigation, multiple samples were prepared for testing. Five specimens were prepared and tested for each composite configuration under tensile, compressive, flexural, and water absorption tests. The reported mechanical properties represent the arithmetic mean values obtained from the five tested specimens for each condition.

The composite preparation involved systematically varying the fiber composition to produce three stepwise functionally graded laminate configurations, as detailed in Table 4. The samples are labeled with specific abbreviations: FH_GS1, FH_GS2, and FH_GS3. Here, “FH” stands for “Functional Hybrid,” whereas “GS” refers to “Glass and Sisal”. The numbers denote the varying levels of sisal content in each formulation.

In composite fabrication, the constituent proportions were initially determined using weight fractions, as presented in Table 4, because weighing provides a practical and accurate approach during manufacturing. However, the analytical and numerical formulations require volume fractions, since the mechanical response of composite materials is governed by the volumetric distribution of the constituents. Therefore, the constituent weight fractions were converted into volume fractions using the densities of the respective materials according to Equations (2) and (3). The calculated overall volume fractions for each composite configuration were subsequently used to determine the layer-wise volume fractions presented in Section 3.1.1 and employed in the analytical calculations. The volume fractions were calculated as:

(2) $$Volume\;fraction\;\left(V_{f}\right)=\frac{Volume\;of\;constituent}{Total\;volume\;of\;the\;composite},$$

The volume of each constituent was then determined using its mass and density, which were expressed as:

(3) $$Volume=\frac{Mass}{Density},$$

Using the constituent densities and Equations (2) and (3), the overall volume fractions of each composite configuration were determined and subsequently distributed among the five laminate layers according to the adopted stepwise functionally graded architecture for use in the analytical model.

### 2.4. Analytical Method

In this proposed method, the analytical analysis begins with the evaluation of the stiffness variation across the laminate thickness. The stiffness distribution is modeled using a power-law function to represent the continuous variation in elastic modulus through the functionally graded laminate. The elastic modulus at position z is expressed as:

(4) $$E(z)=(1-f(z))E_{G}+f(z)E_{S},$$

where $E_{G}$ and $E_{S}$ are the Young’s moduli of the glass and sisal fibers, respectively, and $f(z)={\left(\frac{z+t/2}{t}\right)}^{2}$ is the power-law function describing the stiffness gradation through the laminate thickness, where z varies from $-\frac{t}{2}$ to $+\frac{t}{2}$ [19]. This function models the variation in elastic modulus across the laminate thickness and is used to predict the mechanical response of the functionally graded composite under applied loading.

In addition, the power-law function provides a continuous theoretical representation of the through-thickness variation in elastic modulus. However, the fabricated laminate consists of five discrete layers with equal thickness. Therefore, the continuous stiffness distribution is approximated using a five-layer stepwise configuration in the analytical formulation and numerical simulation, thereby representing the functionally graded behavior of the developed composite.

#### 2.4.1. Strength Evaluation

The strength of the functionally graded hybrid laminate is determined using the stress–strain relationship:

(5) $$\left[\begin{array}{c} \sigma_{x}\\ \sigma_{y}\\ \tau_{xy} \end{array}\right]=\left[\begin{array}{ccc} Q_{11} & Q_{12} & 0\\ Q_{12} & Q_{22} & 0\\ 0 & 0 & Q_{66} \end{array}\right]\left[\begin{array}{c} \in_{x}\\ \in_{y}\\ \gamma_{xy} \end{array}\right],$$

The tensile (or compressive) strength is determined using the Tsai–Hill failure criterion:

(6) $$\frac{{\sigma_{x}}^{2}}{{\sigma_{x}}^{2}}+\frac{{\sigma_{y}}^{2}}{{\sigma_{T}}^{2}}-\frac{\sigma_{x}\sigma_{y}}{{\sigma_{T}}^{2}}\leq 1,$$

where $\sigma_{T}$ is the tensile or compressive strength of each lamina, $\sigma_{x}$ and $\sigma_{y}$ are the normal stresses in the x and y directions, respectively, and k refer to the layer number.

For each layer, the stress $\sigma_{x}(k)$ is calculated based on force equilibrium related to the applied load per unit width $N_{x}$. The failure strength $\sigma_{T}$ is derived from the material properties, allowing for the evaluation of failure conditions [20].

#### 2.4.2. Volume Fraction Distribution

The layer-wise volume fractions used in the analytical calculations were obtained from the overall composite volume fractions calculated from the experimentally measured constituent weight fractions using Equations (2) and (3). These overall volume fractions were subsequently distributed among the five laminate layers according to the adopted stepwise functionally graded architecture.

The distribution of volume fractions across the five discrete layers of the stepwise functionally graded laminate is essential for achieving the desired mechanical properties. Each of the five layers occupies one-fifth (20%) of the total laminate thickness, thereby representing the stepwise approximation of the through-thickness gradation.

The layers are strategically arranged as follows:

1. Glass fiber layers (L1 & L5): These layers are composed of glass fibers and epoxy, serving as the outer reinforcement to provide strength and rigidity;
2. Hybrid layers (L2 & L4): These layers consist of a 50:50 ratio of glass and sisal fibers blended with epoxy, combining the benefits of both materials to enhance mechanical performance while maintaining lightweight characteristics;
3. Sisal core layer (L3): This layer contains primarily sisal fibers with epoxy, providing a natural and lightweight core that contributes to the overall structural integrity.

For each composite type, the overall volume fractions are divided equally among the five layers:

(7) $$V_{GF}=\frac{V_{G}}{2};\;V_{H1}=V_{H}=\frac{V_{H}}{2};\;V_{SF}=V_{S},$$

This systematic approach ensures each layer contributes effectively to the overall mechanical performance.

The calculations for layer-wise volume fractions are based on the overall volume fractions obtained for each composite type:

1. Glass Fiber Layers (L1 & L5): Each layer contains a specified fraction of glass and epoxy;
2. Hybrid Layers (L2 & L4): These layers contain a blend of glass and sisal fibers, ensuring that both materials contribute to the mechanical performance;
3. Sisal Core Layer (L3): This layer primarily consists of sisal fibers, providing lightweight reinforcement [21].

This methodical approach allows for a comprehensive understanding of how each material contributes to the overall performance of the composite, ensuring that the mechanical properties are optimized for structural applications.

#### 2.4.3. Elastic Moduli Calculation

The elastic modulus for each layer is determined using the established volume fractions, using the formula:

(8) $$E_{layer}=V_{f}E_{f}+V_{m}E_{m},$$

where $E_{f}$ is the Young’s modulus of fiber (glass/sisal/hybrid), $V_{f}$ is the volume fraction of fiber in the layer, $E_{m}$ is the Young’s modulus of the matrix (epoxy resin), and $V_{m}=1-V_{f}$ is the volume fraction of the matrix [21].

#### 2.4.4. Tensile Stress Calculation

The tensile stress distribution in each layer is calculated using Hooke’s Law to evaluate the effect of different material compositions on the mechanical performance of the functionally graded composites:

(9) $$\sigma_{Tx}\left(z\right)=E\left(z\right)\varepsilon_{x},$$

where E(z) is the elastic modulus at position z, and $\varepsilon_{x}$ is the applied axial strain, which is calculated as:

(10) $$\varepsilon_{x}=\frac{N_{x}}{\sum_{k=1}^{5}(E_{k}t_{k})},$$

where $N_{x}$ is the applied axial load per unit width, $E_{k}$ is the elastic modulus of the k-th layer, and $t_{k}$ is the thickness of the corresponding layer.

Since the laminate consists of five equal-thickness layers, the thickness of each layer is defined as $t_{k}=t/5$, where t is the total laminate thickness. Therefore, the laminate stiffness term can be expressed as:

(11) $$\sum_{k=1}^{5}(E_{k}t_{k})=\frac{t}{5}\sum_{k=1}^{5}E_{k},$$

By substituting Equation (11) into Equation (10), the average axial strain in the laminate can be expressed as:

(12) $$\varepsilon_{x}=\frac{5N_{x}}{\;tE_{total}},$$

where $E_{total}=\sum_{k=1}^{5}E_{k}$ is the summation of the elastic moduli of all five laminate layers. Equation (12) is the average axial strain developed in the functionally graded laminate under the applied tensile loading condition.

The tensile stress for each layer is calculated as:

(13) $$\sigma_{Tx}=E\left(z\right).\varepsilon_{x},$$

The ultimate tensile strength ($\sigma_{T}$) for a layer can be calculated using the Rule of Mixtures:

(14) $$\sigma_{T,\;layer}=V_{f}\sigma_{T,f}+V_{m}\sigma_{T,m},$$

where $V_{f}$ is the volume fraction of the fiber, $\sigma_{T,f}$ is the ultimate tensile strength of the fiber, $V_{m}=1-V_{f}$ is the volume fraction of the matrix, and $\sigma_{T,m}$ is the ultimate tensile strength of the matrix [22].

#### 2.4.5. Initial Failure Evaluation

The initial failure is evaluated using the Tsai–Hill criterion:

(15) $${\left(\frac{\sigma_{Tx,k}}{\sigma_{T},k}\right)}^{2}\geq 1,$$

This assessment identifies any layers that may have reached their failure limit under applied loads.

### 2.5. Numerical Method

**Figure 6:**
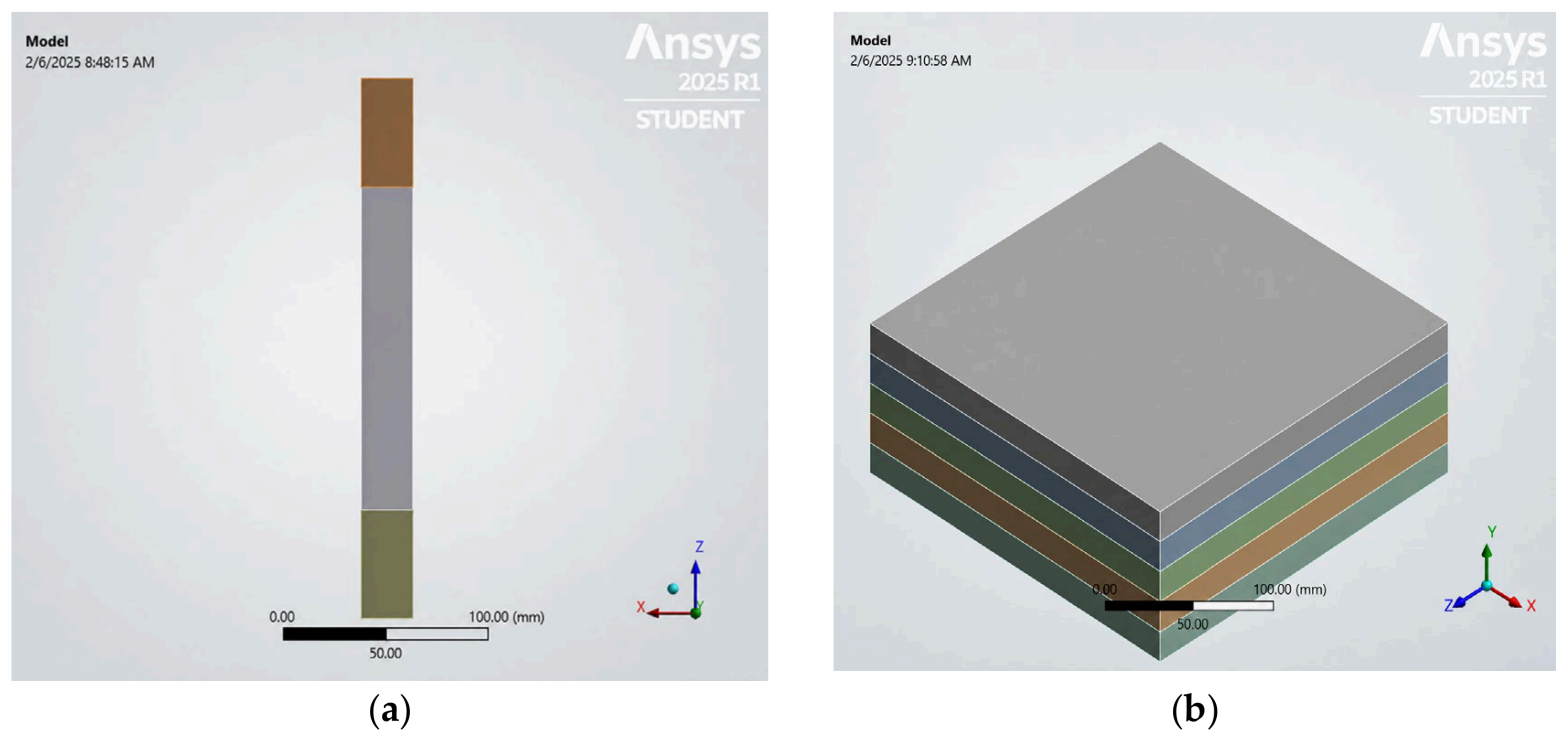
Composite models for: (**a**) tensile sample; (**b**) compression sample.

Finite element simulations were conducted using ANSYS 2025 Student to evaluate the tensile and compressive strengths of the developed functionally graded sisal/glass fiber-reinforced polymer composites. The geometric dimensions of the numerical specimen models were selected to be consistent with the experimental specimens, which were prepared according to the corresponding ASTM-based experimental specimen specifications. Figure 6a shows the tensile specimen model, and Figure 6b presents the compression specimen model used in the numerical analysis. As shown in Figure 6b, the finite element model consists of five layers arranged in the following sequence: epoxy resin, hybrid glass/sisal fiber, epoxy resin, hybrid glass/sisal fiber, and epoxy resin. This stacking sequence represents the laminate configuration adopted in the numerical analysis and is consistent with the functionally graded composite investigated experimentally. The hybrid glass/sisal fiber layers represent the combined reinforcement used in the fabricated laminate, ensuring consistency between the experimental composite configuration and the finite element model.

**Figure 7:**
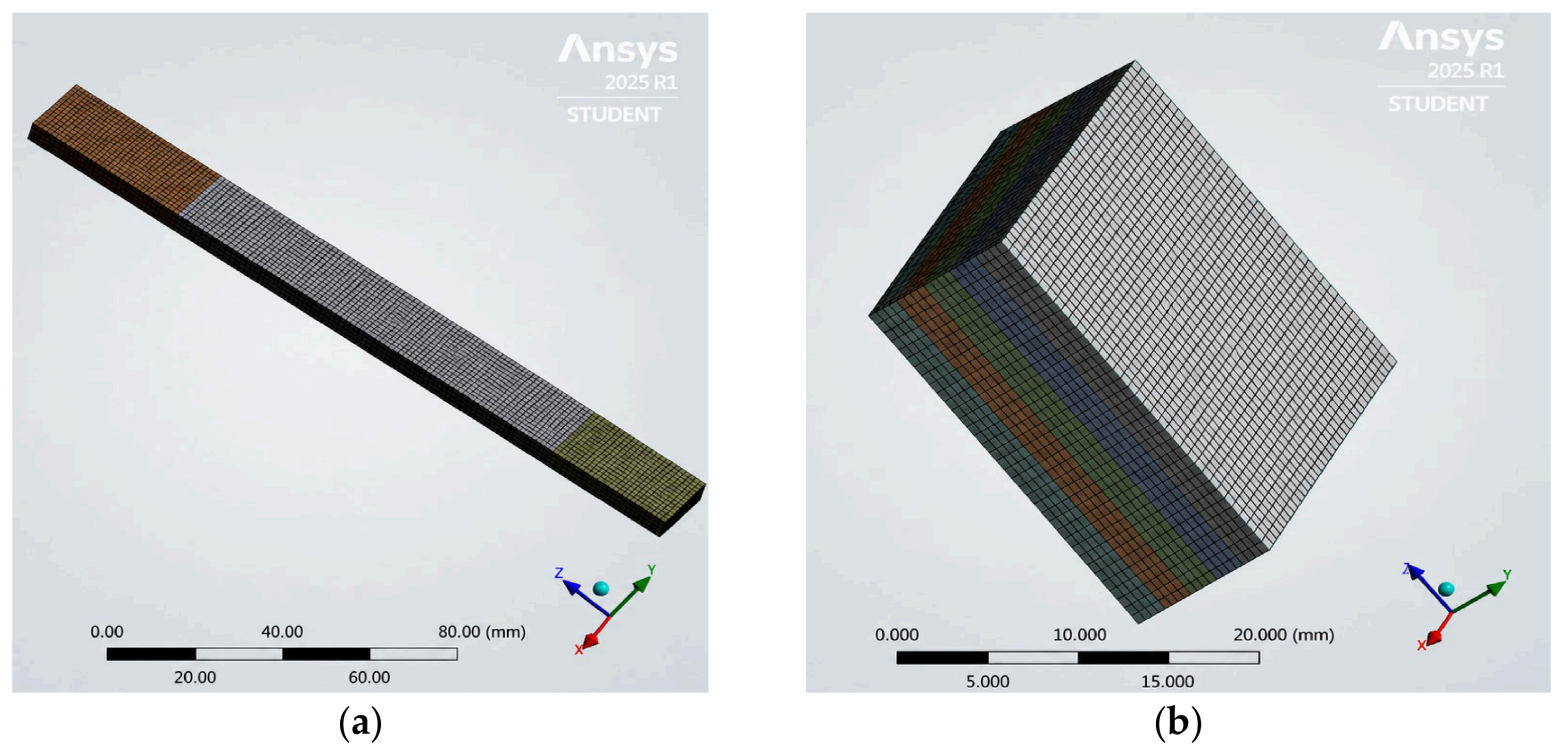
Meshed composite samples: (**a**) tensile sample; (**b**) compression sample.

Meshing was performed on the generated composite models, as shown in Figure 7. A linear meshing approach was employed, resulting in a total of 19,600 elements and 89,229 nodes for the tensile specimen, as depicted in Figure 7a. Figure 7b shows the compression specimen, which comprises 25,920 elements and 112,073 nodes.

**Figure 8:**
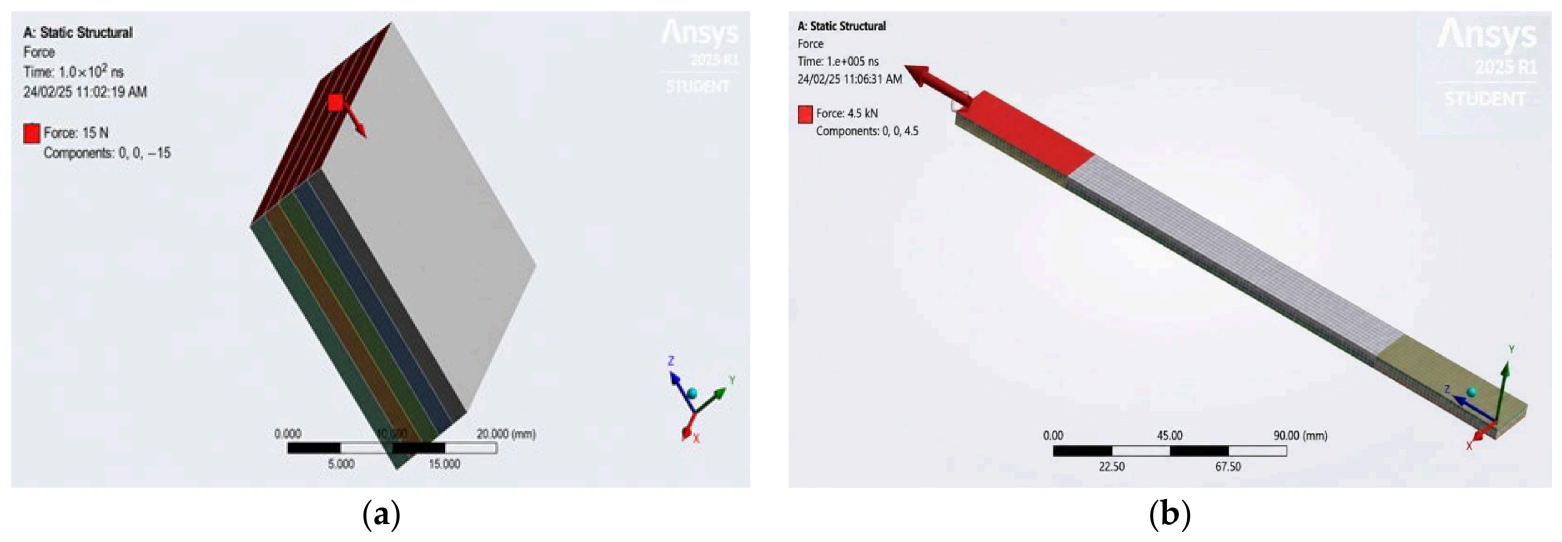
Boundary conditions: (**a**) tensile sample; (**b**) compression sample.

One face of the tensile sample is fixed, while the opposite face is subjected to a tensile force of 4.5 kN in the X-direction, as shown in Figure 8a. Figure 8b shows the compression sample, which has a force of 15 kN applied.

### 2.6. Mechanical Testing

Following the fabrication process, the mechanical properties of the developed functionally graded hybrid composites were assessed through mechanical tests. These tests included tensile strength, compression, and flexural testing, all conducted at room temperature to ensure consistency and reliability of the results.

The specimen dimensions, gripping arrangements, loading configurations, and test procedures were selected according to the requirements of the relevant ASTM standards. Tensile testing was conducted following ASTM D3039 for rectangular specimens [23] and ASTM D7205 for rod-shaped specimens [24], compression testing was performed according to ASTM D3410 [25], and flexural testing was conducted following ASTM D790 [26]. These standards were considered during specimen preparation and testing to ensure appropriate geometry, fixture arrangement, and loading conditions throughout the experimental investigation.

#### 2.6.1. Tensile Strength Tests

**Figure 9:**
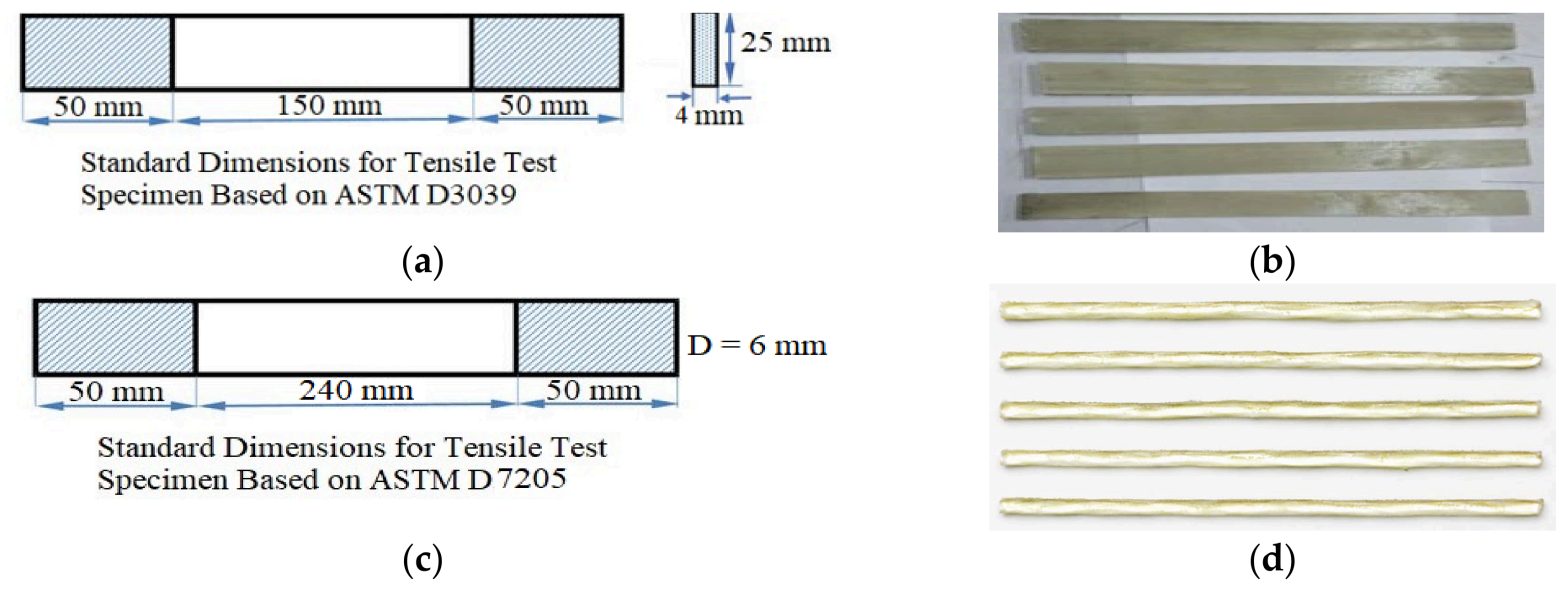
Tensile strength test specimens: (**a**) dimensions of rectangular specimens; (**b**) photograph of rectangular specimens; (**c**) dimensions of rod-shaped specimens; (**d**) photograph of rod specimens.

Tensile strength tests were conducted following ASTM D3039 for rectangular specimens [23] and ASTM D7205 for rod-shaped specimens [24]. The objective was to evaluate the tensile performance of the developed composites. For each composite composition, five rectangular specimens and five rod-shaped specimens were prepared, as shown in Figure 9. Testing these conventional shapes provides a comprehensive assessment of the tensile properties of the material across different geometries.

**Figure 10:**
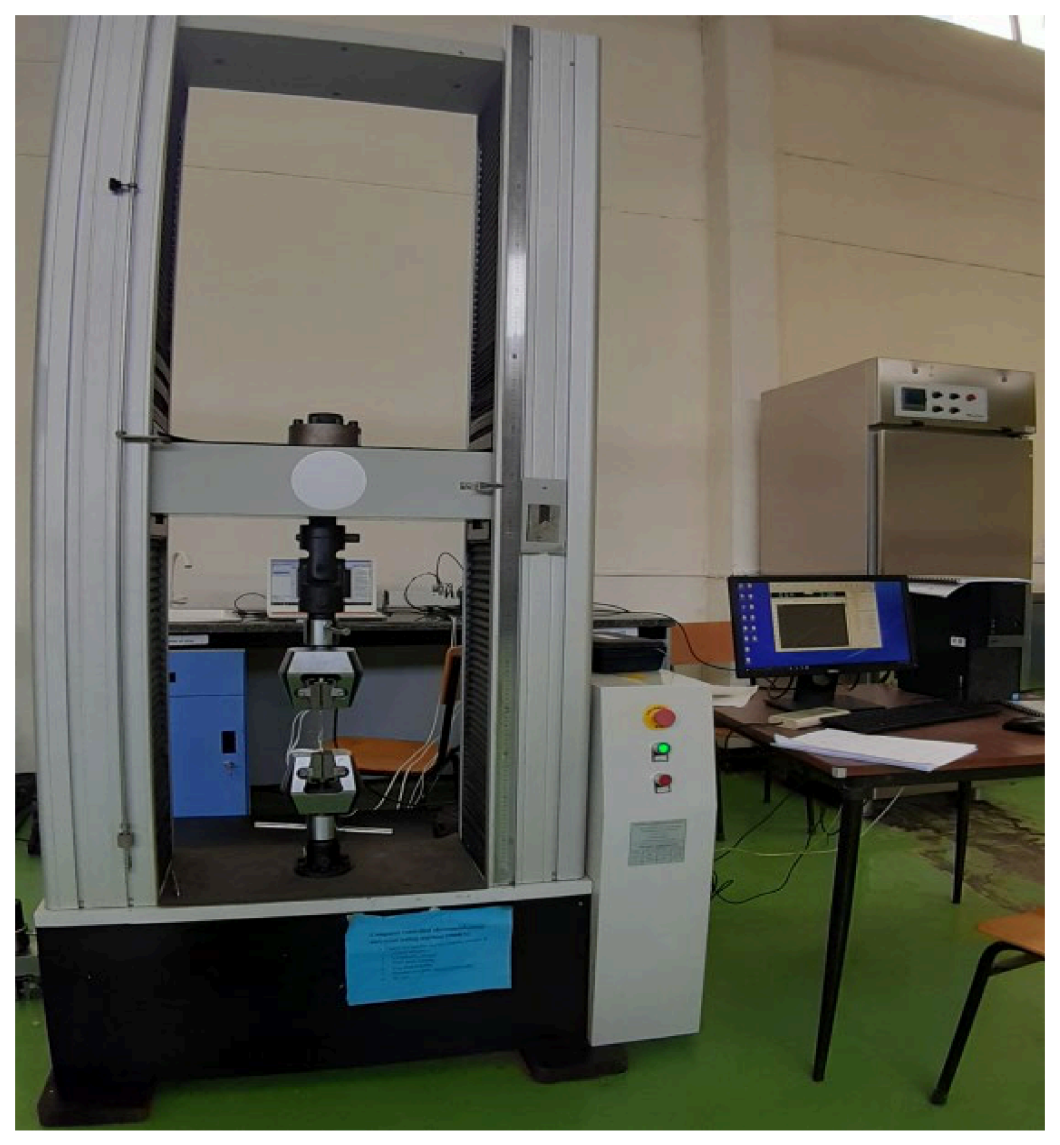
Photograph of the tensile testing machine.

The prepared specimens were secured in the grips of a computer-controlled electromechanical UTM, which has a maximum capacity of 100 kN. The experiments were carried out with a crosshead speed set to 2 mm/min in displacement control mode. A photograph of the testing machine is illustrated in Figure 10.

During the testing process, load–displacement curves were continuously recorded. From these graphs, the strength and elongation at break were calculated. The tensile strength results were expressed in megapascals (MPa), providing a clear measure of the performance of the developed material under tensile loading conditions.

#### 2.6.2. Compressive Strength Tests

**Figure 11:**
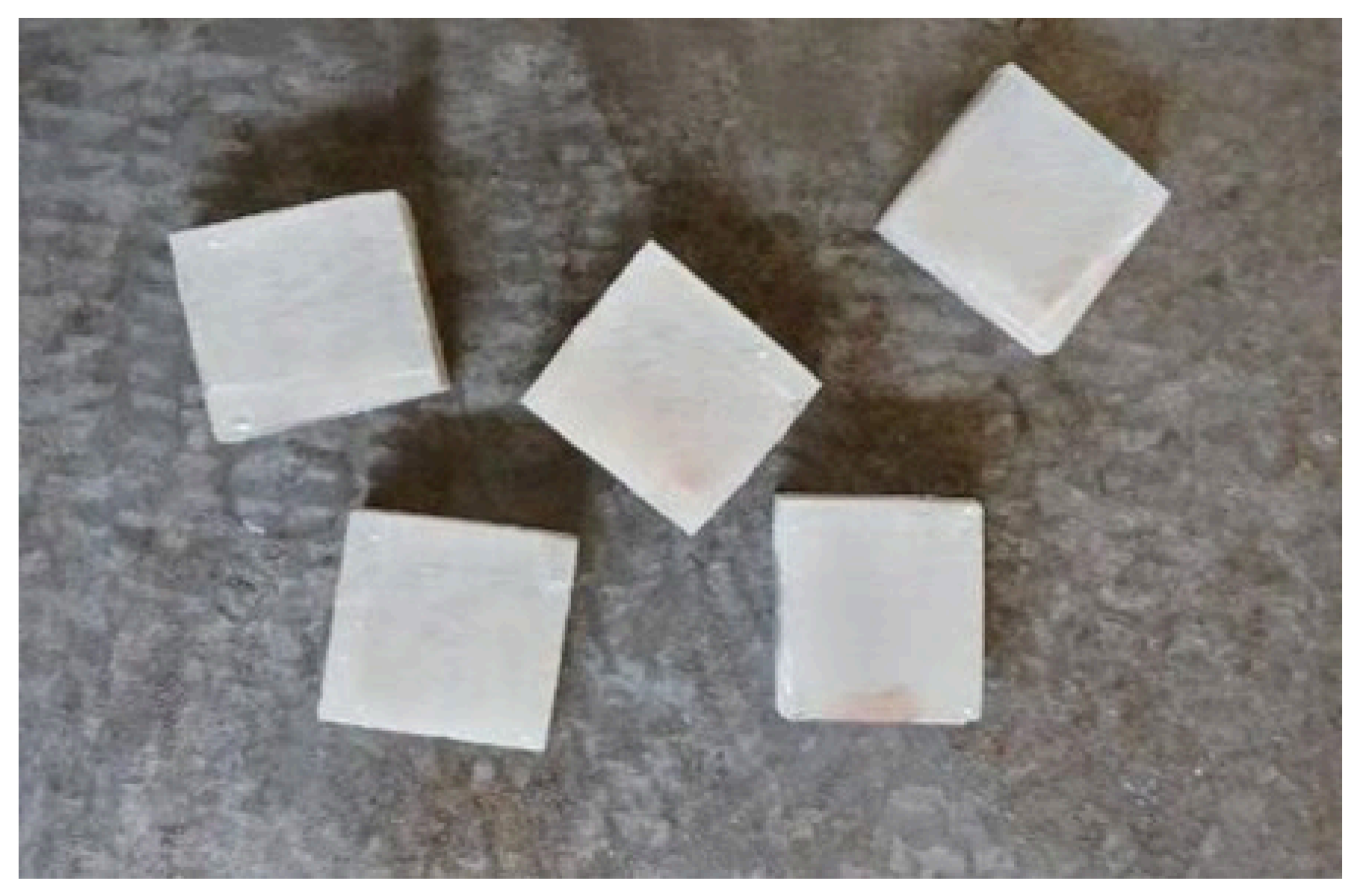
Photograph of compression strength test samples.

The strength tests were conducted on the functionally graded sisal/glass fiber-reinforced polymer composites to evaluate their performance under compressive loads. For this study, five rectangular samples (25 mm × 25 mm × 10 mm) were prepared for each composite composition based on the ASTM D3410 standard [25]. Figure 11 shows the photograph of the test specimens of the hybrid composite that were utilized in the compression tests.

The compression tests were carried out using a computer-controlled electromechanical UTM equipped with a 100 kN load cell. The machine allowed for controlled testing conditions, which is crucial for obtaining consistent results. The tests were performed with the crosshead speed maintained at 2 mm/min in displacement control mode. This testing speed was chosen to minimize dynamic effects and ensure that the samples were subjected to a gradual increase in load.

During the tests, the load–deformation curves were carefully recorded. From the load–deformation data, the compressive strength was calculated.

#### 2.6.3. Flexural Strength Tests

**Figure 12:**
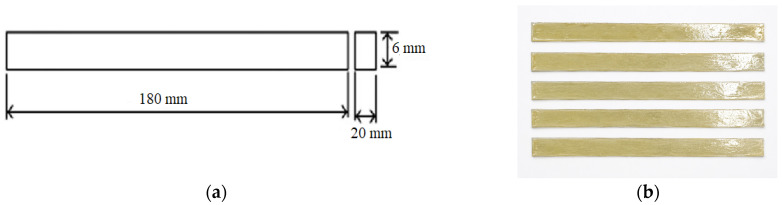
Flexural strength test specimens: (**a**) standard specimen dimensions; (**b**) photograph of the test specimens.

Flexural strength experiments were performed to evaluate the performance of functionally graded sisal/glass fiber-reinforced polymer composites under bending loads. Rectangular beam-shaped specimens (180 mm × 20 mm × 6 mm) were prepared, based on ASTM D790 [26]. The gauge length for the test was set to 96 mm as stated in the standard. The standard dimensions and the photographs of the test samples are shown in Figure 12.

The flexural experiments were performed at ambient temperature using a three-point bending configuration. The flexural experiments were performed using a computerized UTM equipped with a capacity of 100 kN. The specimens were supported at two points, whereas a point load was employed at the center of the beam until fracture occurred. The speed of the machine crosshead was set to 2 mm/min.

The testing was conducted at Addis Ababa Science and Technology University (AASTU) in Addis Ababa, Ethiopia. During the test, the load-deflection curve was continuously recorded. From the recorded data, the flexural parameters were calculated. The flexural strength is reported in MPa.

#### 2.6.4. Water Absorption Test

The mechanical properties of the developed materials can be significantly affected by moisture absorption, leading to a reduction in their overall performance. To evaluate the water absorption behavior of the developed composites, a water absorption test was performed based on the ASTM D5229 standard [27], which is designed to determine the moisture absorption or desorption properties of polymer matrix composite materials [27].

**Figure 13:**
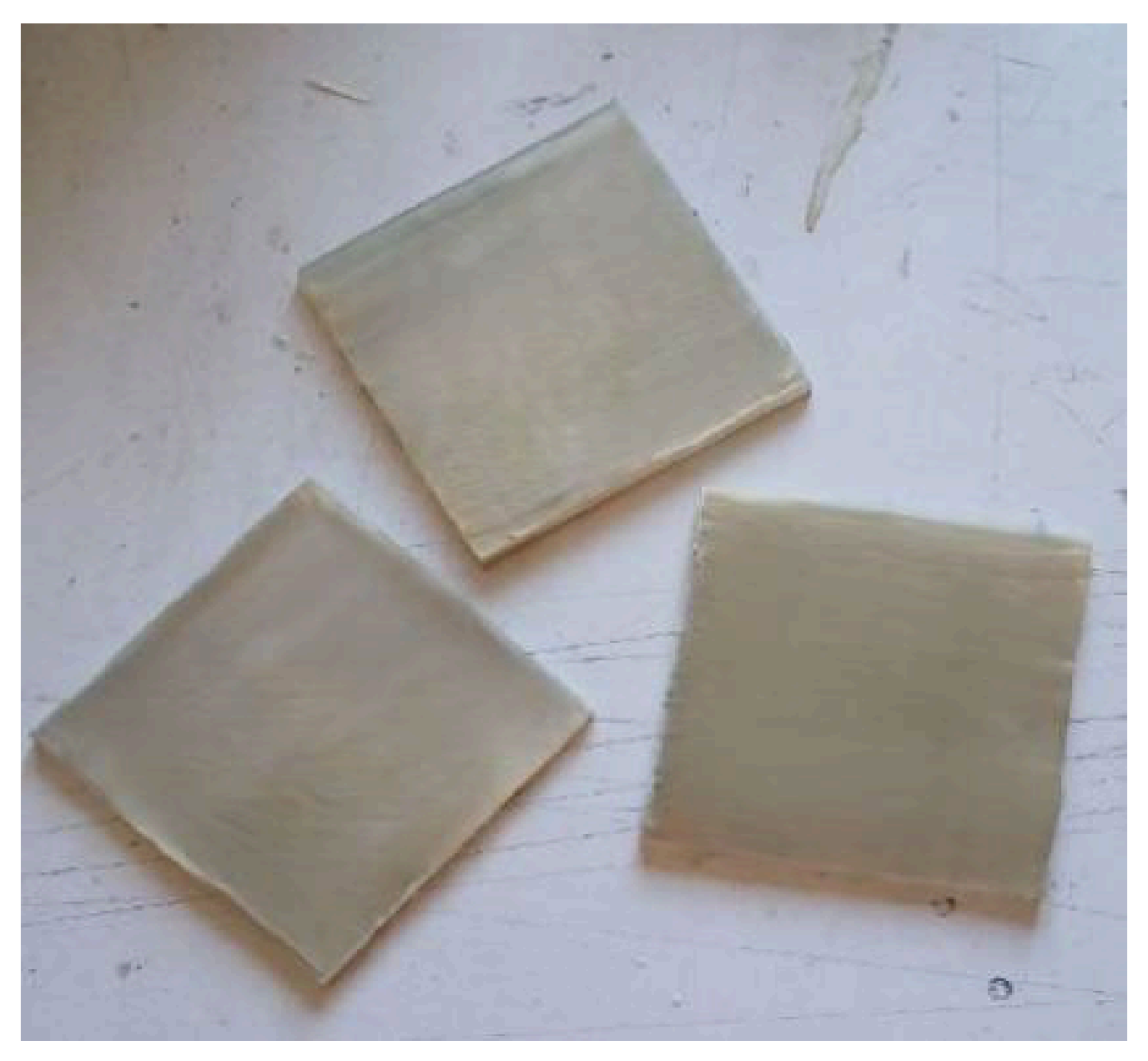
Photograph of water absorption test specimens.

For this test, composite specimens were prepared in square shapes. A total of five test specimens for each composite were used, each measuring 100 mm × 100 mm. The edges of the samples were covered with resin, which can prevent moisture absorption through the sides, ensuring that the measurement focused solely on the exposed surfaces. The photograph for the water absorption test specimen from each composite is presented in Figure 13.

The testing process began with drying the specimens, which were then left to cool naturally. Once cooled, each specimen was measured using an analytical balance with a precision of 0.001 mg to determine its initial weight ($W_{o}$). The specimens were then submerged in distilled water at room temperature for 24 h, and their weight was recorded afterward ($W_{t}$) to assess water absorption capacity.

To capture the initial phase of absorption, the specimens were weighed at regular intervals, particularly after the first 12 h, and subsequently every 72 h until saturation was reached. The material was considered saturated when the weight increase did not exceed 1% of the previous measurement or when the absorbed weight change was less than 0.1 g between weighings. The water absorption percentage was determined as:

(16) $$\mathrm{Water}\;\mathrm{absorption}\;(\%)=\frac{W_{t}-W_{o}}{W_{o}}\times 100,$$

where $W_{o}$ is the initial dry weight of the specimen before immersion and $W_{t}$ is the weight after immersion. The water absorption percentage is calculated relative to the initial dry weight, providing a quantitative measure of the moisture absorbed by the composite specimens.

## 3. Results and Discussion

This section presents and analyzes the results obtained from experimental investigations, FEA, and theoretical evaluations of the developed functionally graded sisal/glass fiber-reinforced polymer composites. Firstly, the mechanical properties of the composites are investigated through experimental testing and numerical simulations. Experimental investigations of water absorption behavior are also presented to evaluate the influence of fiber content on moisture uptake. Furthermore, a comparison with conventional materials based on existing literature is provided, followed by a comprehensive discussion of the findings.

### 3.1. Analytical Results

#### 3.1.1. Volume Fraction Distribution Results

For each composite type (FH_GS1, FH_GS2, FH_GS3), the total volume fraction is divided equally among the five layers, ensuring a balanced distribution of composite constituents. The resulting layer-wise volume fractions are detailed in Table 5, Table 6 and Table 7.

As seen from these results, the structured distribution of volume fractions across layers is a critical aspect of achieving optimized mechanical performance in functionally graded composites. In FH_GS1, the glass fiber layers (L1 & L5) dominate the composition, providing significant rigidity due to their high epoxy content. The hybrid layers (L2 & L4) introduce a moderate amount of glass fiber, which enhances interlayer bonding and improves mechanical properties. The sisal layer (L3), although containing a smaller percentage of glass, adds a natural fiber component that contributes to sustainability while also improving toughness. In FH_GS2, there is a noticeable increase in the volume fraction of sisal fibers in layer L3, which elevates the hybridization effect. This change will lead to improved energy absorption characteristics, making the composite more resilient under dynamic loading conditions. For FH_GS3, the increased sisal content in layer L3 significantly enhances the composite’s natural fiber properties while maintaining a balanced epoxy matrix. This configuration improves tensile performance while maintaining a lightweight structure, which is advantageous for weight-sensitive applications. This careful arrangement of materials ensures that the composite exhibits desirable mechanical properties, such as increased stiffness, strength, and toughness, while also addressing sustainability concerns. The findings suggest that functionally graded composites with optimized volume fractions can effectively meet the demands of various structural applications, paving the way for their integration into advanced engineering solutions.

#### 3.1.2. Elastic Moduli Summary

The elastic moduli for each layer across the composite types are summarized in Table 8.

The variation in elastic moduli across the different composite types illustrates the impact of material combinations on the stiffness of each layer. Notably, the sisal core layer in FH_GS3 exhibits a significant increase in elastic modulus, reaching 8.219 GPa. This enhancement suggests improved load-bearing capacity and structural integrity, making it particularly advantageous for applications requiring high strength and durability.

**Figure 14:**
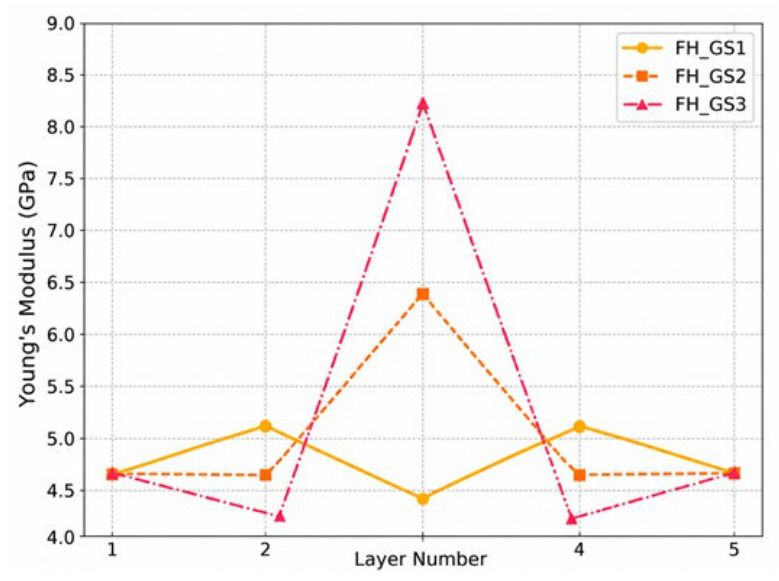
Variation in elastic moduli across layers.

The stiffness variation across layers is depicted in Figure 14. The analysis of elastic moduli highlights the importance of material selection and layer arrangement in determining the mechanical performance of functionally graded composites. The consistent values for the glass layers (L1 & L5) across all composite types indicate their stability in providing rigidity and strength. The hybrid layers (L2 & L4) exhibit variability in elastic modulus, emphasizing the influence of fiber composition on performance. The decrease in elastic modulus for FH_GS2 and FH_GS3 in the hybrid layers suggests that while sisal fibers contribute to toughness, they also reduce stiffness compared to glass fibers. The pronounced increase in the elastic modulus of the sisal core layer in FH_GS3 signifies that optimizing natural fiber content can yield substantial benefits in terms of mechanical properties. This finding underscores the potential of sisal fibers to enhance the overall performance of composites while promoting sustainability.

#### 3.1.3. Tensile Stress Results

The tensile stress results for each layer across the composite types (FH_GS1, FH_GS2, and FH_GS3) are summarized in Table 9.

The values indicate the tensile stress experienced by each layer under the applied load, reflecting how the composition affects the mechanical performance. The hybrid layers (L2 & L4) consistently show higher tensile stresses compared to the glass and sisal core layers, indicating their enhanced ability to withstand tensile forces. Notably, the sisal core layer in FH_GS3 exhibits the highest tensile stress (78.0 MPa), suggesting a significant improvement in performance due to the increase in sisal fiber content.

The maximum tensile stresses at initial failure for the various composite compositions are presented in Table 10.

These results indicate the maximum tensile stress that each layer can withstand before failure. The glass layers (L1 & L5) maintain relatively consistent values across the composites, providing reliable strength. However, the hybrid layers show a decline in maximum tensile stress as the sisal content increases, particularly in FH_GS3, likely due to the natural fiber’s lower tensile strength compared to glass. In contrast, the sisal core layer in FH_GS3 demonstrates a remarkable increase (159.78 MPa) in maximum tensile stress at initial failure. This suggests that the incorporation of sisal fibers enhances the overall load-bearing capacity of the composite, especially when optimized within the layer structure.

**Figure 15:**
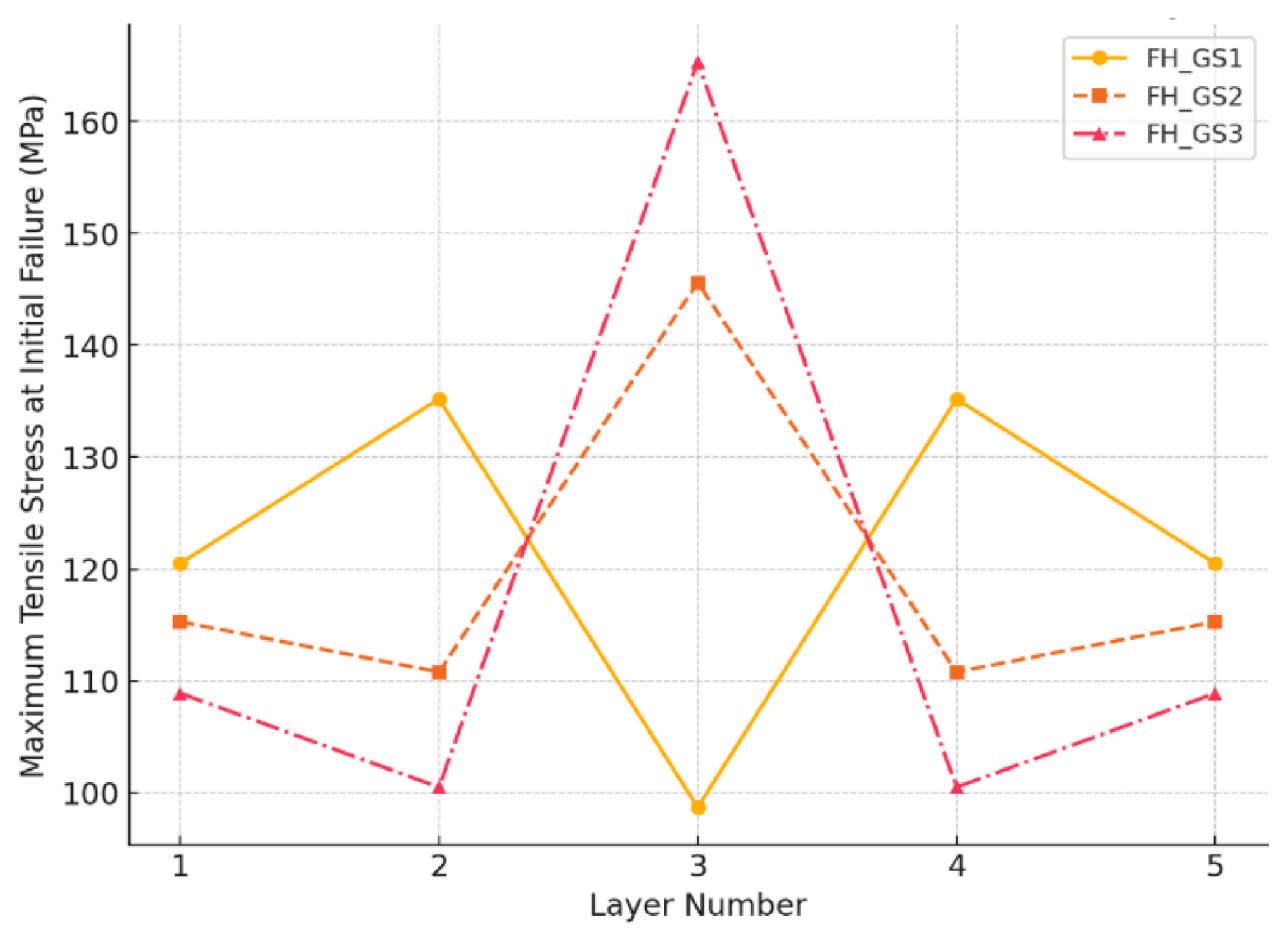
Maximum tensile stress at initial failure across layers.

Figure 15 shows the maximum tensile stress at initial failure across the different layers of FH_GS1, FH_GS2, and FH_GS3.

The tensile stress results provide valuable insights into the mechanical behavior of functionally graded composites. The consistently higher tensile stresses in the hybrid layers indicate their superior performance under tensile loading, attributed to the synergistic effects of combining glass and sisal fibers. The significant increase in maximum tensile stress at initial failure for the sisal core layer in FH_GS3 highlights the potential of natural fibers to enhance composite performance. This finding reinforces the notion that optimizing fiber composition can lead to improved mechanical properties, making these composites suitable for structural applications where strength and weight considerations are critical.

#### 3.1.4. Initial Failure Evaluation Using Tsai–Hill Criterion

The results of the initial failure evaluation using the Tsai–Hill criterion are summarized in Table 11.

All layers across the three composite types (FH_GS1, FH_GS2, and FH_GS3) exhibit failure indices below 1, indicating that no layers have reached their failure limits under the applied loads. This result suggests that the composites maintain structural integrity and can withstand operational stresses effectively.

**Figure 16:**
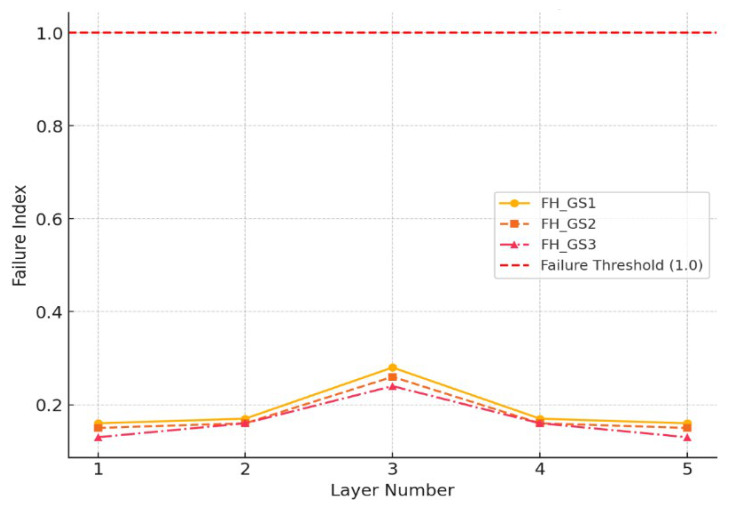
Variation in the failure index across layers of FH_GS1, FH_GS2, and FH_GS3.

Figure 16 shows the variation in the failure index across the layers for the different composite types.

The failure index results from the Tsai–Hill criterion further confirm the robustness of the functionally graded composites. The consistent “Pass” outcomes across all layers indicate that the engineered design successfully prevents failure under anticipated loading conditions. The failure indices are relatively low, reflecting a significant margin of safety in the composite structures. This safety margin implies that the composites can endure unexpected loads or stresses beyond typical operational conditions. The variation in failure indices across the layers also highlights the effectiveness of the layered material arrangement. Each layer’s contribution to the overall performance ensures that the composites can leverage the strengths of both natural and synthetic fibers, thereby enhancing overall durability and reliability. Hence, the results from the Tsai–Hill criterion support the viability of using functionally graded sisal/glass fiber-reinforced composites in structural applications, reaffirming their potential for high-performance engineering solutions.

### 3.2. Numerical Results

**Figure 17:**
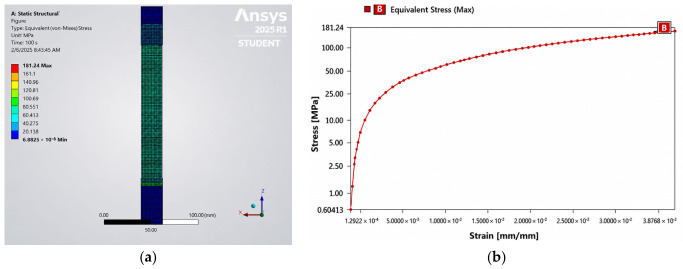
Equivalent tensile stress results: (**a**) tensile strength; (**b**) stress–strain curve.

In this work, FEA was also performed to investigate the tensile and compressive behavior of the functionally graded sisal/glass fiber-reinforced polymer composites. Figure 17a shows the tensile strength of the composite at an applied force of 4.5 kN. The FEA results indicate that the maximum tensile strength of the composite is 181.24 MPa, while the experimental results show a tensile strength of 146 MPa. This discrepancy is attributed to variations in material properties, manufacturing processes, or testing conditions. In addition, Figure 17b presents the stress–strain curve, which illustrates the relationship between stress and strain during the tensile test. This curve provides valuable insights into the material’s behavior under load, highlighting its capacity for deformation before failure.

**Figure 18:**
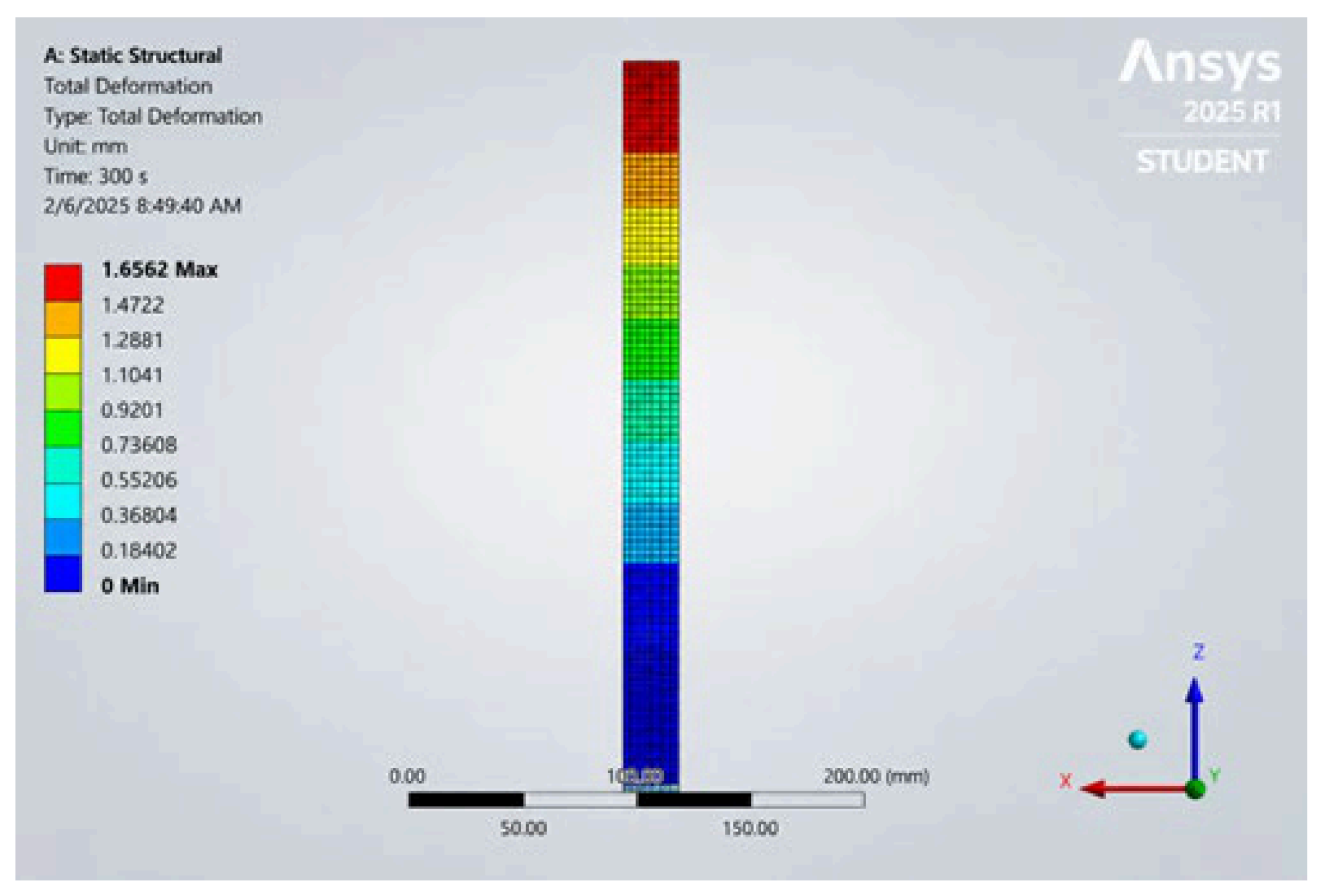
Total deformation of the composite under tensile loading.

As shown in Figure 18, the total deformation of the composite under tensile loading is measured at 1.6562 mm. This deformation reflects the material’s ability to undergo significant strain, which is crucial for evaluating its performance and suitability for various applications.

**Figure 19:**
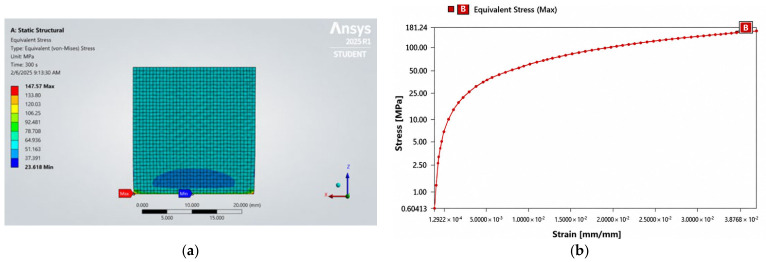
Equivalent compressive stress results: (**a**) compressive strength; (**b**) stress–strain curve.

Similarly, Figure 19a displays the compressive strength of the composite under an applied force of 15 kN. The simulations indicate a maximum compressive strength of 147.57 MPa, whereas the experimental results show a compressive strength of 120 MPa. This difference arises from variations in material properties, manufacturing processes, or testing conditions. Figure 19b also presents the stress–strain curve, which illustrates the relationship between stress and strain during the compressive test. This curve provides important insights into the material’s behavior under compressive loading, emphasizing its capacity to withstand deformation before failure.

**Figure 20:**
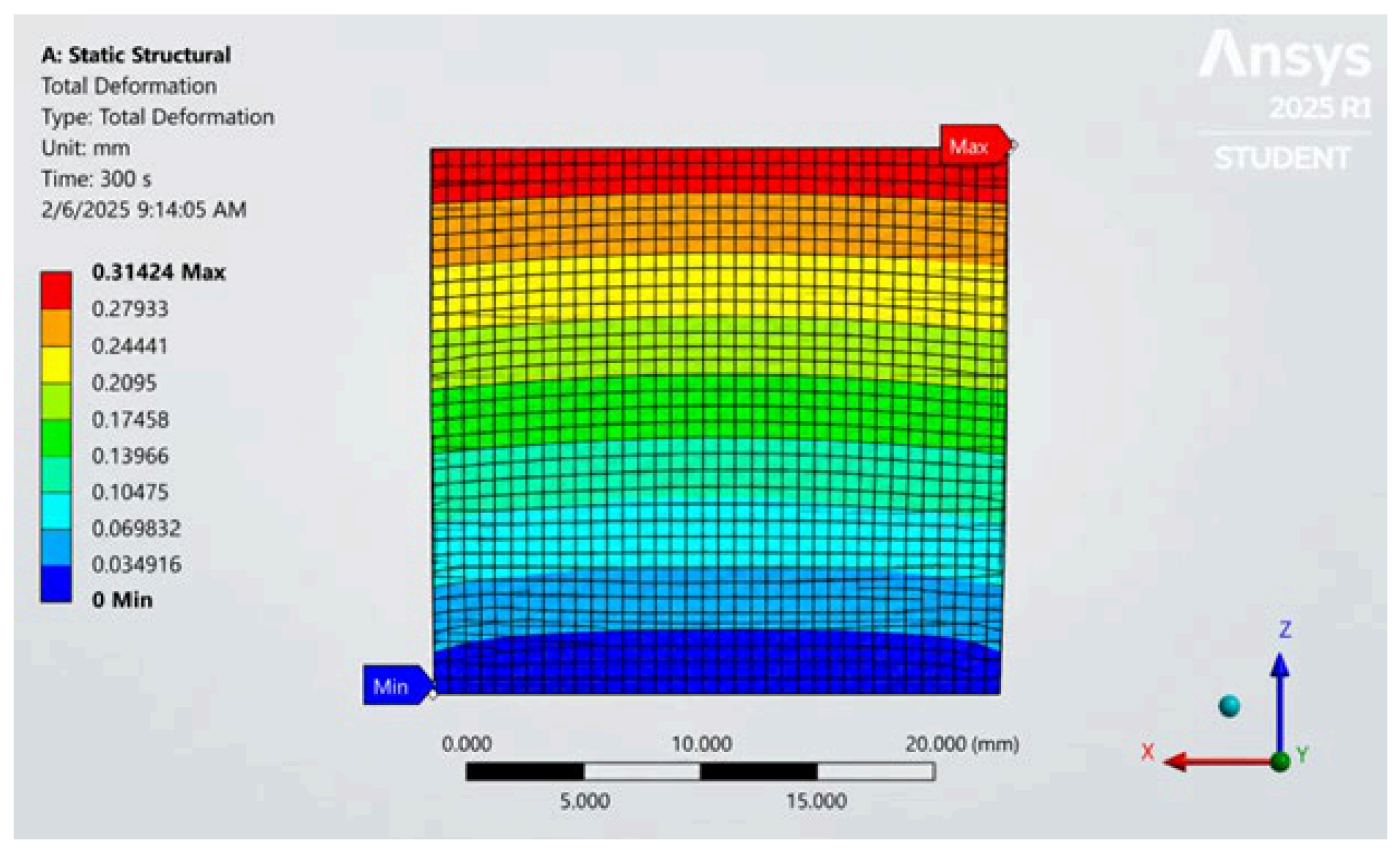
Total deformation of the composite under compressive loading.

As shown in Figure 20, the total deformation of the composite under compressive loading is measured at 0.31424 mm. This deformation indicates the material’s ability to undergo significant strain, which is essential for assessing its performance and suitability for various applications.

In general, the numerical simulations conducted for both tensile and compressive tests provide valuable insights into the mechanical properties of the functionally graded sisal/glass fiber-reinforced polymer composites. The simulations show that the tensile and compressive strengths exceed the experimental values, suggesting potential for optimization in composite design. The stress–strain curves for both tensile and compressive tests reveal the material’s ability to deform significantly before failure, indicating good ductility and resilience. Thus, the findings highlight the composites’ promising performance characteristics, which can be further enhanced through controlled manufacturing processes and material property improvements. These results serve as a foundation for future research and applications in various engineering fields.

### 3.3. Experimental Results

#### 3.3.1. Tensile Strength

**Figure 21:**
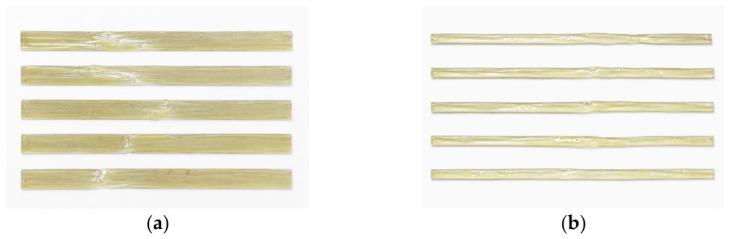
Specimens after tensile testing: (**a**) rectangular specimen; (**b**) rod-shaped specimen.

The tensile strength of the developed functionally graded composites was evaluated to assess the effect of sisal fiber content. Tensile strength, defined as the maximum stress a material can endure before necking occurs, is crucial for structural applications. Figure 21 presents the specimens after the tensile test, showcasing the fractured specimens.

**Figure 22:**
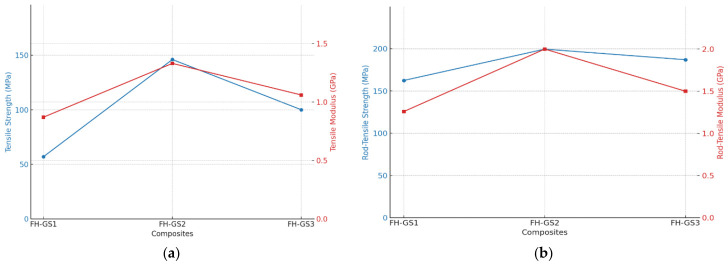
Tensile strength of the composite: (**a**) influence of sisal fiber content on tensile strength for rectangular shapes; (**b**) influence of sisal fiber content on tensile strength for rod shapes.

Figure 22 shows the effects of sisal fiber content on the tensile strength of the material for both rectangular and rod shapes.

Figure 22a shows that the tensile strength of the rectangular specimens increased significantly with increasing sisal fiber content. The tensile strength values recorded are 57 MPa for specimens with 10% sisal fibers, 146 MPa for those with 20%, and 100 MPa for specimens reinforced with 30% sisal fibers. These results indicate that incorporating sisal fibers enhances the load-bearing capacity of the composite. Nevertheless, the low tensile strength observed at 10% sisal fiber indicates that this reinforcement level is insufficient, resulting in suboptimal structural performance.

Similarly, for rod-shaped specimens, as presented in Figure 22b, the tensile strengths also exhibit a positive correlation with increased fiber content. The tensile strength values for these specimens are 162.5 MPa at 10% sisal, 199.5 MPa at 20% sisal, and an impressive 187 MPa at 30% sisal. These results further confirm that higher sisal fiber content contributes positively to its strength. However, beyond a certain amount of fiber content, the strength decreases; this indicates the effect of fiber clustering and void formation as the natural fiber content increases. A comparison of the two specimen geometries revealed that the rod-shaped specimens consistently exhibited higher tensile strength. This observation indicates that the rod shape offers improved load distribution and stress management, rendering it more advantageous for structural applications where strength is crucial. The enhanced mechanical performance of rod-shaped specimens underscores the importance of optimizing both fiber content and specimen shape in the development of materials.

#### 3.3.2. Compression Strength

**Figure 23:**
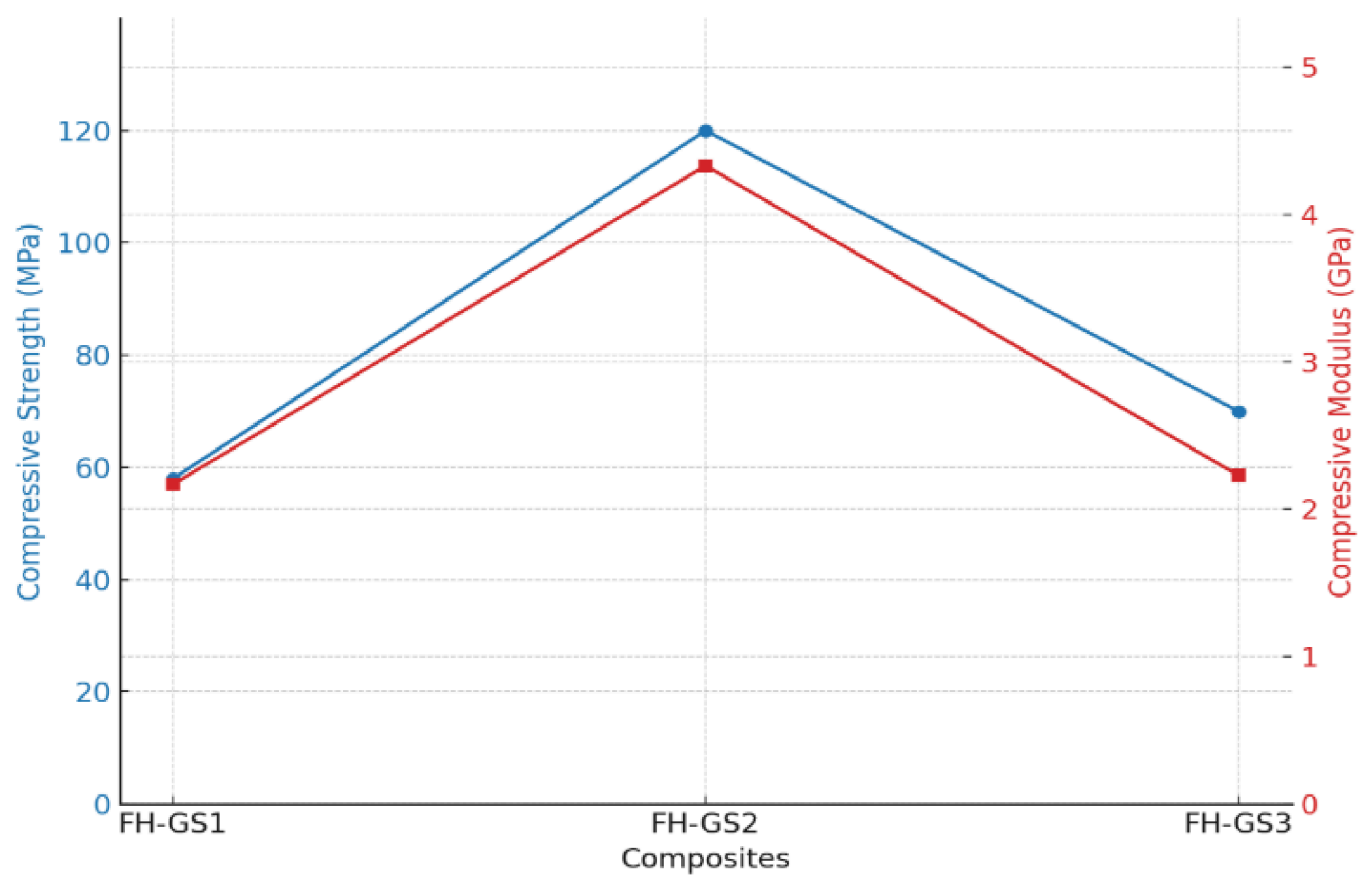
Compression strength of the composite at varying sisal fiber contents.

The resistance of the developed functionally graded composites to compressive forces was assessed by evaluating their compression strength. Figure 23 presents the compression strength results for the composites at varying sisal fiber contents.

From Figure 23, the compression strength of the composites shows considerable variation with the amount of sisal fiber added. Specifically, the recorded compression strength values are as follows: 58 MPa for specimens with 10% sisal fiber, 120 MPa for those with 20% sisal fiber, and 70 MPa for specimens reinforced with 30% sisal fiber. This trend indicates that higher sisal fiber content improves the material’s resistance to compressive forces, with the 20% fiber content level exhibiting the greatest strength. The notable increase in compression strength from 10% to 20% sisal fiber suggests that this range is optimal for achieving improved mechanical performance. However, the slight decrease in compression strength observed at 30% sisal fiber indicates a potential issue with fiber clustering or inadequate bonding within the composite matrix, which can lead to reduced effectiveness in load transfer during compression.

Comparing these results to those obtained from tensile strength tests, it is clear that while both tensile and compressive strengths benefit from increased content of sisal fiber, the optimal fiber content differs. The maximum compression strength observed at 20% sisal fiber content highlights the importance of balancing fiber content to achieve superior mechanical properties in composite materials.

#### 3.3.3. Flexural Strength

**Figure 24:**
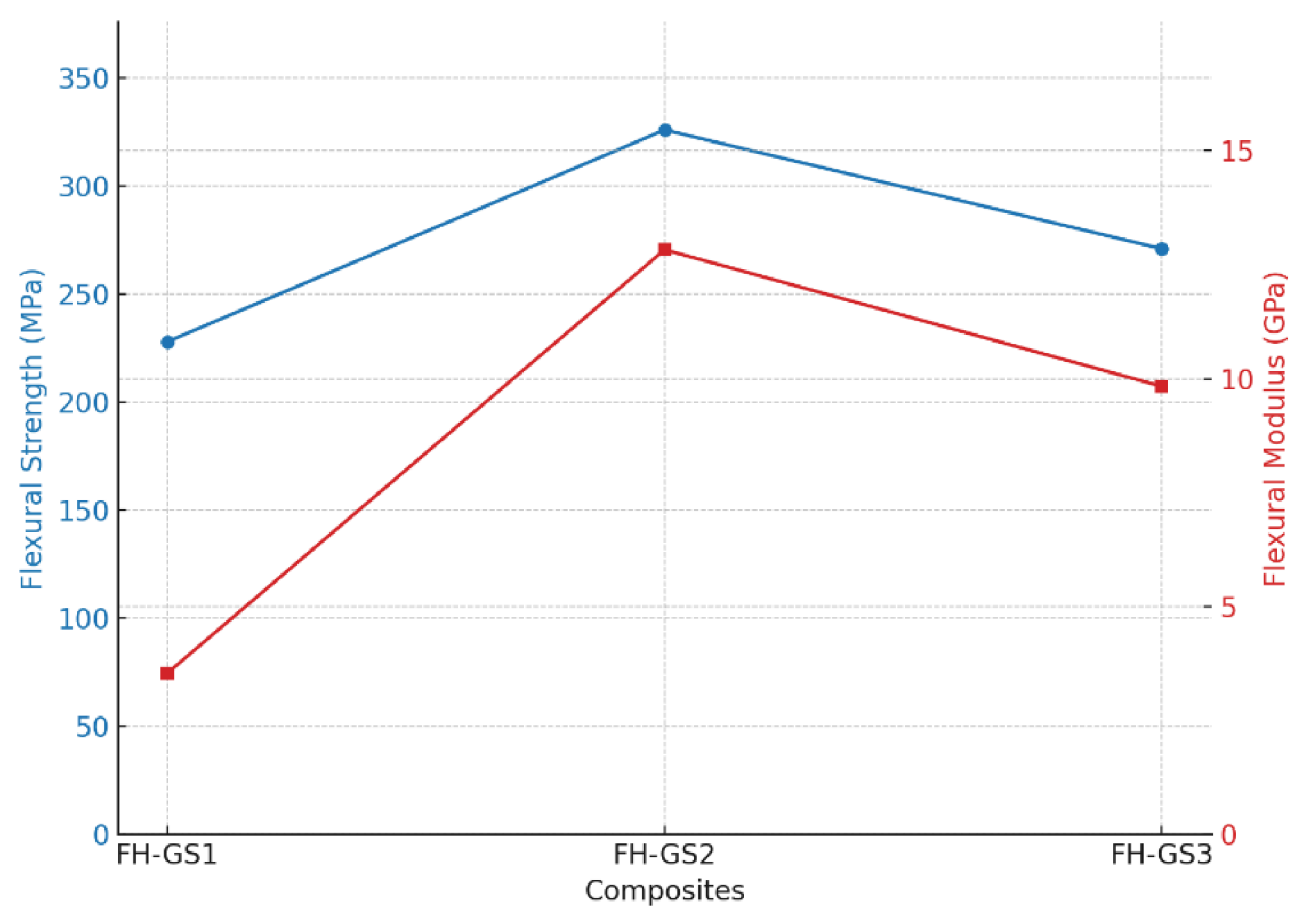
Flexural strength of the composite at varying sisal fiber contents.

Flexural strength represents a critical mechanical property that reflects a material’s ability to withstand bending or flexural stresses without failure. This property plays a significant role in applications subjected to bending loads, as it directly contributes to maintaining structural integrity and reliability. Figure 24 shows the flexural strength of the developed composites.

The findings presented in Figure 24 reveal that the flexural strength of the composites varies significantly with the content of sisal fiber. The recorded flexural strength values are 226 MPa for specimens containing 10% sisal fiber, 326 MPa for those with 20% sisal fiber, and 271 MPa for specimens reinforced with 30% sisal fiber. This data indicates a clear trend: flexural strength increases with the addition of sisal fiber, and attains its peak at 20% fiber content. The substantial increase in flexural strength from 10% to 20% sisal fiber suggests that this level of reinforcement optimally enhances the resistance of the material to bending stresses. However, the decline in flexural strength observed at 30% sisal fiber content indicates a potential compromise in performance at higher fiber concentrations. This decrease is due to various factors, such as fiber clumping, weakened bonding between the fibers and the epoxy matrix, or an imbalance in the composite structure, all of which can impede effective load distribution.

When comparing the flexural strength results to those from tensile and compressive strength tests, these results indicate that while the addition of sisal fiber enhances mechanical properties across the board, the optimal fiber content for flexural strength differs from that of tensile and compressive strengths.

### 3.4. Comparison Analysis with Other Materials

To evaluate the mechanical performance of the developed composite, a comparison with previously reported materials was conducted. This analysis highlights how the functionally graded sisal/glass fiber-reinforced composites outperform several conventional materials and other existing composites in terms of mechanical strength. Table 12 provides the results of this comparative analysis, showcasing the mechanical properties of the developed functionally graded composites alongside those of conventional materials and other composites.

The mechanical properties of the developed functionally graded hybrid composite demonstrate notable improvements compared to other natural and hybrid fiber composites. With a tensile strength of 199.5 MPa, the composite outperforms several traditional hybrid materials, such as jute-glass (185 MPa) and flax/jute fiber composites (90 MPa). While it does not match the tensile strength of metals like mild steel (350–450 MPa) or aluminum alloys (310–570 MPa), its performance highlights its viability as a sustainable alternative in lightweight applications. In terms of flexural strength, the composite achieves an impressive 326 MPa, surpassing hybrid sisal/glass composites (190 MPa) and Sisal and bamboo hybrid composites (137.50 MPa), and approaching the flexural performance of epoxy-based sisal/glass hybrids (411.43 MPa). The compressive strength of the composite, recorded at 120 MPa, is comparable with sisal-bamboo fiber composites (120.08 MPa) but remains lower than traditional engineering materials, reflecting some limitations of polymer matrices under compression.

**Figure 25:**
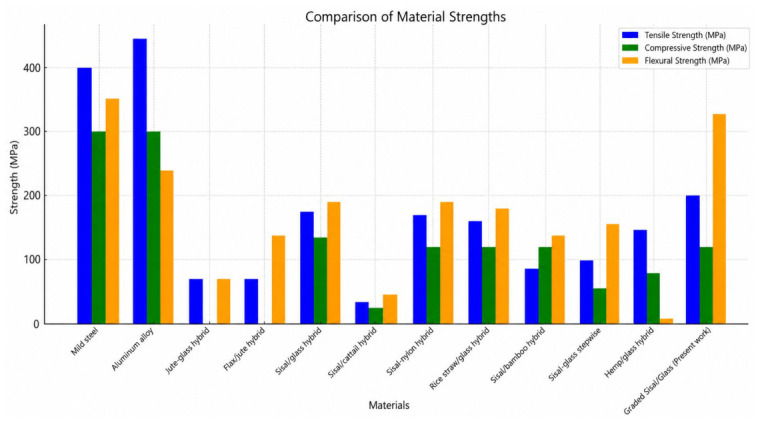
Comparison of mechanical properties of the developed material with existing materials.

To better illustrate the comparison results, Figure 25 presents the tensile, compressive, and flexural strengths of the developed functionally graded material alongside existing materials. As shown in Figure 25, the mechanical properties of the developed functionally graded material compare favorably to those of conventional materials.

The application of stepwise functional gradation significantly enhances load distribution and stress management, contributing to the composite’s enhanced mechanical properties. Compared to similar research, such as the sisal/glass hybrid with gradation (98.26 MPa tensile, 156 MPa flexural), the developed composite demonstrates marked improvements. Furthermore, when compared to hemp-glass hybrid rebar (145.86 MPa tensile, 8.46 MPa flexural), the advantages in tensile and flexural performance are evident, particularly for structural uses where bending resistance is critical. These findings highlight the potential of the developed composite for environmentally conscious structural applications, offering a promising balance of strength, sustainability, and lightweight design.

The mechanical performance observed in the developed stepwise functionally graded composite demonstrates the potential of the proposed layered architecture for structural applications. Compared with several natural and hybrid fiber-reinforced composites reported in the literature, the developed composite exhibited higher tensile and flexural strengths while maintaining the sustainability advantages associated with natural fiber reinforcement. However, because conventional non-graded laminates with the same constituent materials were not fabricated and experimentally evaluated in the present study, the observed improvements cannot be attributed exclusively to the stepwise functional grading. Instead, the results demonstrate the feasibility and promising performance of the proposed laminate configuration. Future studies should include direct experimental comparisons between equivalent stepwise graded and non-graded laminates to quantitatively evaluate the specific contribution of the graded architecture to the overall mechanical performance.

### 3.5. Water Absorption Characteristics

The water absorption behavior of the developed composites is examined in this section, with particular emphasis on the influence of varying fiber contents on moisture uptake. The corresponding results are presented in Table 13 and Table 14.

The results reveal distinct trends in water absorption among the different composite formulations. FH-GS1 exhibited the lowest water absorption rates throughout the testing period, attributed to its lower sisal fiber content and a higher degree of glass fiber encapsulation within the matrix. This configuration effectively minimizes the exposure of hydrophilic sisal fibers, thereby enhancing the moisture resistance of the composite. In contrast, FH-GS2 displayed moderate water absorption levels, correlating with the increased content of sisal fiber. The presence of more sisal fibers introduces additional hydrophilic sites, allowing for greater moisture uptake compared to FH-GS1, while still maintaining reasonable performance. Conversely, FH-GS3 exhibited the greatest water absorption, reflecting the inherent hydrophilic nature of sisal fibers, especially at elevated concentrations. This significant moisture uptake underscores the challenges associated with using higher levels of natural fibers, which can compromise the overall durability and performance of the composite.

**Figure 26:**
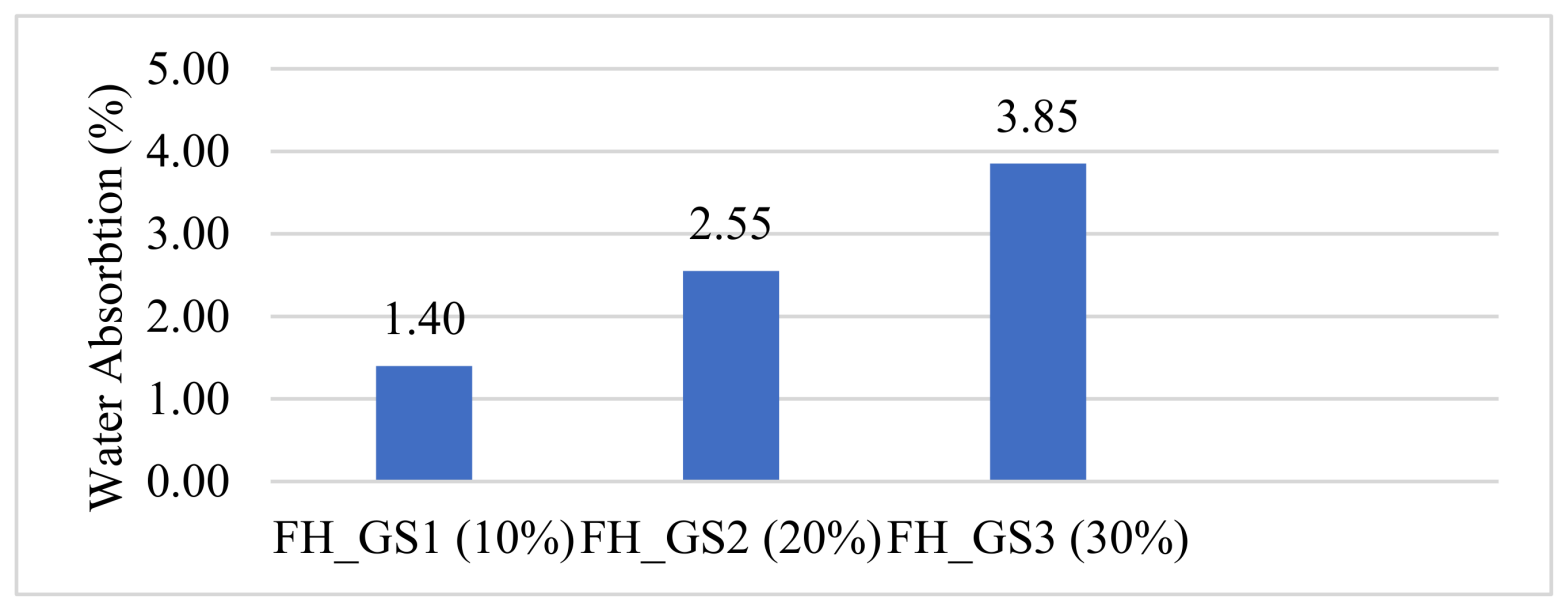
Water absorption characteristics.

To provide a clearer understanding of the water absorption behavior for the three fiber contents, Figure 26 shows the water absorption results of the composites with different sisal fiber contents.

From Figure 26, the water absorption of the composites varies with the amount of sisal fiber incorporated. The recorded water absorption values are 1.40% for specimens containing 10% sisal fiber, 2.55% for those with 20% sisal fiber, and 3.85% for specimens reinforced with 30% sisal fiber. This trend indicates that increasing the sisal fiber content leads to higher moisture uptake, particularly at the 30% fiber content level, which demonstrates the highest absorption. The notable increase in water absorption from 10% to 20% sisal fiber suggests that while moderate fiber content allows for reasonable moisture resistance, the significant rise at 30% indicates potential challenges associated with higher natural fiber concentrations.

**Figure 27:**
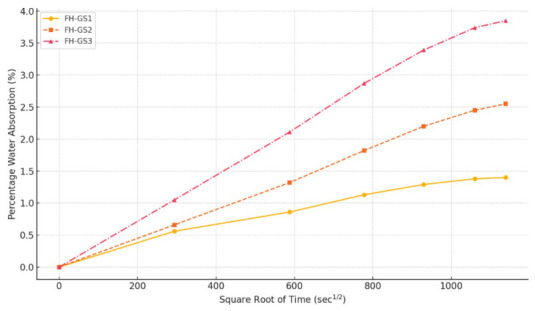
Percentage of water absorption as a function of the square root of time.

The percentage of water absorption of the developed functionally graded composites is plotted against the square root of time, as shown in Figure 27. The plots reveal that higher sisal fiber content significantly enhances moisture uptake, particularly at the 30% level. The increase in water absorption can be attributed to the hydrophilic nature of sisal fiber, the formation of voids, and a greater interfacial area between the fiber and the matrix. In contrast, moisture absorption by epoxy is almost negligible due to its hydrophobic nature.

### 3.6. Discussions

The mechanical properties obtained through experimental testing align with trends predicted by both theoretical models and numerical simulations, though experimental deviations highlight the influence of real-world fabrication challenges. The FEA predicted a tensile strength of 181.24 MPa, which is slightly higher than the 146 MPa obtained experimentally. This deviation can be attributed to fabrication inconsistencies, such as fiber misalignment, void formation, and interfacial debonding, which were not accounted for in the idealized simulation model. Similarly, compressive and flexural strengths followed the expected trends but exhibited lower experimental values due to the occurrence of natural fiber defects.

The Tsai–Hill failure criterion, applied to predict failure initiation, showed reasonable agreement for fiber volume fractions up to 20% but underestimated failure onset at higher fiber contents. This discrepancy suggests that additional factors, such as fiber waviness and non-uniform stress distribution, influence failure mechanisms in real-world applications. Furthermore, elastic modulus calculations based on the Rule of Mixtures closely matched experimental values at low fiber contents but deviated at higher sisal fiber fractions, indicating matrix-dominated deformation effects.

Therefore, the combined experimental, analytical, and numerical approaches provide a comprehensive understanding of the mechanical behavior of the developed composites. Refining numerical models to incorporate real-world imperfections, such as fiber misalignment and interfacial effects, is essential for improving predictive accuracy.

The mechanical properties of the developed material were thoroughly examined, starting with tensile strength. A clear correlation was observed between increasing sisal fiber content and enhanced tensile strength, especially in rectangular specimens, where tensile strength reached 57 MPa at 20% sisal reinforcement. This finding supports a previous paper that indicates higher fiber content improves load-bearing capacity and structural performance. In addition, rod-shaped specimens consistently exhibited higher tensile strengths compared to rectangular ones, suggesting that geometry plays a significant role in load distribution and stress management, which are crucial for structural applications.

Compression strength results further highlighted the impact of sisal fiber content, with optimal performance achieved at 20% fiber content, resulting in a maximum strength of 120 MPa. This trend indicates that moderate fiber content enhances resistance to compressive forces, aligning with literature that suggests excessive fiber can lead to clustering and reduced bonding [39]. The decline in compression strength observed at 30% sisal content illustrates potential challenges in load transfer within the composite matrix. Flexural strength results mirrored these findings, peaking at 20% sisal fiber content with a recorded strength of 326 MPa, followed by a decrease at 30%. The increase in flexural strength with the addition of sisal fibers underscores its significance in applications subjected to bending stresses. Conversely, the reduction at higher concentrations suggests that factors such as fiber clumping and inadequate bonding hinder effective load distribution.

Moreover, the assessment of water absorption characteristics is vital for understanding the durability of these composites. High moisture uptake can lead to diminished mechanical performance, particularly in humid environments. The observed water absorption behavior is associated with both the fiber content and the applied alkaline treatment. However, since untreated reference fibers were not investigated in the present study, the individual contribution of alkaline treatment to moisture resistance and mechanical performance cannot be quantitatively separated. This consideration is particularly relevant for structural applications, where moisture management is critical for maintaining mechanical integrity over time.

The results indicate that the developed stepwise functionally graded composite results in mechanical properties comparable to or superior to several natural and hybrid fiber-reinforced composites reported in the literature, highlighting its potential for structural applications. Nevertheless, the findings should be interpreted considering certain limitations. The fabricated specimens represent a five-layer stepwise functionally graded architecture rather than a continuously graded material, and equivalent non-graded laminates were not evaluated for direct comparison. Therefore, the specific contribution of the grading architecture cannot be quantitatively isolated. Additionally, although alkaline treatment was applied to improve sisal fiber–matrix compatibility based on previous studies, its direct contribution was not experimentally quantified in the present work. The analytical and numerical models also adopted a stepwise approximation and did not explicitly incorporate manufacturing imperfections, such as fiber misalignment, voids, and interfacial effects. Future studies should include direct comparisons with non-graded laminates, untreated fiber composites, and refined models incorporating fabrication-induced effects to further quantify the influence of grading architecture and fiber surface treatment.

## 4. Conclusions

This study successfully developed and characterized functionally graded sisal/glass fiber-reinforced polymer composites, demonstrating their potential for structural applications. To accomplish this, an experimental approach was employed, focusing on the characterization of mechanical properties and the assessment of water absorption behavior. The following findings are drawn from the analysis.

The detailed analysis of the mechanical properties revealed a clear correlation between sisal fiber content and enhanced performance. The composite’s tensile strength showed a significant increase with higher sisal fiber content, reaching 146 MPa for the rectangular cross-section and 199.5 MPa for the composite bar at 20% sisal fibers. In addition, compressive and flexural strengths peaked at 120 MPa and 326 MPa, respectively, at an optimal 20% sisal fiber content. This indicates that an optimal fiber concentration exists for maximizing mechanical performance, while excessive fiber content will lead to diminishing returns in strength.

The geometric configuration of the specimens was found to be critical in influencing mechanical performance. Rod-shaped specimens consistently demonstrated higher tensile strengths compared to rectangular ones, suggesting that geometry plays a significant role in effective load distribution and stress distribution. This finding highlights the significance of considering shape in the design and use of these composites, especially for structural components that need greater load-bearing capacity.

The investigation into water absorption behavior revealed that lower sisal fiber content resulted in significantly reduced moisture uptake, enhancing the composite’s durability. Conversely, higher sisal fiber concentrations led to increased water absorption, reflecting the inherent hydrophilic nature of sisal fibers. This highlights potential durability challenges in high-moisture environments and indicates that careful management of fiber content is essential to mitigate water absorption and maintain mechanical integrity.

In addition to experimental results, theoretical and numerical analyses played a crucial role in validating the findings. FEA yielded maximum tensile and compressive strengths of 181.24 MPa and 147.57 MPa, respectively, showing reasonable agreement with the experimental results. The observed differences are primarily attributed to fabrication imperfections, material heterogeneity, and idealized boundary condition assumptions adopted in the numerical model. The Tsai–Hill failure criterion and Rule of Mixtures provided a theoretical framework for optimizing fiber volume fractions, confirming that a 20% sisal fiber content achieves the best balance between mechanical performance and durability. These results highlight the importance of integrating experimental, numerical, and theoretical approaches, providing a more robust and comprehensive framework for material characterization.

Although this paper successfully developed and characterized functionally graded sisal/glass fiber-reinforced polymer composites, some areas remain open for further investigation:

(1) Microstructural Analysis: A detailed study of fiber–matrix interactions using techniques such as Scanning Electron Microscopy (SEM) and X-ray diffraction (XRD) would provide deeper insights into bonding mechanisms, void distribution, and interfacial adhesion;
(2) Optimization of Fabrication Methods: Exploring automated fabrication techniques such as resin infusion, compression molding, or additive manufacturing (3D printing) could improve composite uniformity, reduce defects, and enhance scalability for industrial applications;
(3) Hybridization with Other Natural Fibers: Investigating the effect of incorporating other natural fibers (e.g., flax, hemp, or kenaf) alongside sisal in functionally graded composites could further optimize mechanical properties and expand potential applications.

Future studies should focus on these aspects to further improve the mechanical performance and durability of functionally graded natural fiber composites.

## Figures and Tables

**Table 1:** Properties of glass fiber provided by the supplier.

Properties	Glass Fiber
Diameter, µm	11.0
Density, g/m^3^	2.5
Poisson’s ratio	0.21–0.23
Elastic modulus, GPa	70.0
Shear modulus, GPa	30–36
Elongation, %	2.5–3.0
Tensile strength, MPa	2000–3000
Coefficient of thermal expansion, $\times 70^{-6}/{}^{\circ}C$	5.0
Thermal conductivity, W/m/°C	1.3

**Table 2:** Characteristics of general-purpose epoxy and hardener.

Characteristics	General-Purpose Epoxy Resin	HY951 Hardener
Type	Bisphenol-A-based general-purpose epoxy resin	Aliphatic amine-based hardener
Viscosity	10,000–12,000 mPa·s at 25 °C	10–20 mPa·s at 25 °C
Density	1.15–1.20 g/cm^3^	0.97–1.00 g/cm^3^
Epoxy equivalent weight	182–192 g/eq	-
Mixing ratio (resin: hardener)	100:10 (by weight)	100:10 (by weight)
Pot life (working time)	-	~30–45 min at 25 °C
Tensile strength (cured)	~60 MPa	-
Flexural strength (cured)	~90 MPa	-
Glass transition temperature	150–170 °C (post-cured)	150–170 °C (post-cured)
Cure time	Gel time: ~2–3 h at 25 °C; Full cure: 24 h	-
Appearance	slightly yellow liquid	light-yellow liquid

**Table 3:** Physical and mechanical properties of sisal fibers [18].

Properties	Sisal Fibers
Type	*Agave sisalana*
Average diameter, mm	0.279
Density, g/m^3^	1.5
Elastic modulus, GPa	20
Tensile strength, MPa	385
Poisson’s ratio	0.3
Shear Modulus, GPa	3

**Table 4:** Composition of constituents in the composites.

Constituents	Low Sisal Content (wt. %)	Medium Sisal Content (wt. %)	High Sisal Content (wt. %)
	FH_GS1	FH_GS2	FH_GS3
Epoxy	72	63	54
Hardener	8	7	6
Glass fibers	10	10	10
Sisal fibers	10	20	30

The fiber content levels (10%, 20%, and 30%) were selected based on existing studies demonstrating that natural fiber composites typically exhibit optimal mechanical properties within this range. Below 10%, the reinforcement is insufficient, while above 30%, processing challenges such as fiber clustering and void formation reduce performance.

**Table 5:** Volume fractions of FH_GS1.

Layer	Glass Fiber $V_{G}$	Hybrid $V_{H}$	Sisal $V_{S}$	Epoxy $V_{m}$
L1 & L5 (Glass)	2.5%	0%	0%	97.5%
L2 & L4 (Hybrid)	0%	5.05%	0%	94.95%
L3 (Sisal)	0%	0%	8.3%	91.7%

**Table 6:** Volume fractions of FH_GS2.

Layer	Glass Fiber $V_{G}$	Hybrid $V_{H}$	Sisal $V_{S}$	Epoxy $V_{m}$
L1 & L5 (Glass)	2.5%	0%	0%	97.5%
L2 & L4 (Hybrid)	0%	3.95%	0%	96.05%
L3 (Sisal)	0%	0%	19.9%	80.1%

**Table 7:** Volume fractions of FH_GS3.

Layer	Glass Fiber $V_{G}$	Hybrid $V_{H}$	Sisal $V_{S}$	Epoxy $V_{m}$
L1 & L5 (Glass)	2.55%	0%	0%	97.45%
L2 & L4 (Hybrid)	0%	3.2%	0%	96.8%
L3 (Sisal)	0%	0%	30.7%	69.3%

**Table 8:** Elastic moduli for each layer.

Layer	FH_GS1 (GPa)	FH_GS2 (GPa)	FH_GS3 (GPa)
L1 & L5 (Glass)	4.675	4.675	4.708
L2 & L4 (Hybrid)	5.121	4.659	4.344
L3 (Sisal Core)	4.411	6.383	8.219

**Table 9:** Tensile stress for each layer.

Layer	FH_GS1 (GPa)	FH_GS2 (GPa)	FH_GS3 (GPa)
L1 & L5 (Glass)	48.7	46.6	44.7
L2 & L4 (Hybrid)	53.3	46.4	41.3
L3 (Sisal Core)	45.9	63.7	78.0

**Table 10:** Maximum tensile stress at initial failure.

Layer	FH_GS1 (GPa)	FH_GS2 (GPa)	FH_GS3 (GPa)
L1 & L5 (Glass)	121	121	122.22
L2 & L4 (Hybrid)	129.82	114.59	104.24
L3 (Sisal core)	86.97	124.68	159.78

**Table 11:** Failure index results.

Layer	FI_FH_GS1	Pass/Fail	FI_FH_GS2	Pass/Fail	FI_FH_GS3	Pass/Fail
1	0.16	Pass	0.15	Pass	0.13	Pass
2	0.17	Pass	0.16	Pass	0.16	Pass
3	0.28	Pass	0.26	Pass	0.24	Pass
4	0.17	Pass	0.16	Pass	0.16	Pass
5	0.16	Pass	0.15	Pass	0.13	Pass

**Table 12:** Mechanical properties comparison of the developed functionally graded composites with existing materials.

Ref.	Materials	Tensile Strength (MPa)	Compressive Strength (MPa)	Flexural Strength (MPa)
Engineering standards	Mild steel	350–450	250–350	300–400
Engineering standards	Aluminum alloy	310–570	200–400	240
Pawar et al. [28]	Natural/synthetic hybrid (jute-glass)	71.29	-	69.37
Benkhelladi et al. [29]	Flax/jute hybrid composite	69.3	-	136.06
Bodduru et al. [30]	Sisal/glass hybrid composite	175	135	190
Mbeche et al. [31]	Sisal/cattail hybrid composites	32.3	24.6	44.8
Hari et al. [32]	Sisal/nylon hybrid composite	170	120	190
Ali-Eldin et al. [33]	Rice straw/glass hybrid Composite	160	120	180
Gupta et al. [34]	Hybrid Sisal/Glass with Epoxy Composite	87.22	-	411.43
Ganta and Patel [35]	Sisal and bamboo hybrid fiber-reinforced composites	85.08	120.08	137.50
Composite-Tech [36]	GFRP Rebar	1300	550	-
Ashuro et al. [37]	Sisal/glass hybrid with stepwise gradation	98.26	55.32	156
Suresha K V et al. [38]	Hemp/glass hybrid reinforced epoxy composite	145.86	79.72	8.46
Present work	Functionally graded Sisal/Glass polymer composite	199.5	120	326

**Table 13:** The average weight of composite materials over time.

Time (Days)	FH-GS1 (g)	FH-GS2 (g)	FH-GS3 (g)
0 (Dry)	102.172	103.252	105.297
1	102.741	103.938	106.407
4	103.052	104.615	107.525
7	103.326	105.135	108.320
10	103.489	105.522	108.870
13	103.582	105.783	109.233
15	103.600	105.890	109.340

**Table 14:** Percentage of water absorption over time.

Time (Days)	FH-GS1 (%)	FH-GS2 (%)	FH-GS3 (%)
0 (Dry)	0.00	0.00	0.00
1	0.56	0.66	1.05
4	0.86	1.32	2.11
7	1.13	1.82	2.87
10	1.29	2.20	3.39
13	1.38	2.45	3.74
15	1.40	2.55	3.85

## Data Availability

The original contributions presented in this study are included in the article. Further inquiries can be directed to the corresponding authors.
